# Complex Contributions of Methylglyoxal to Pain, Axon Degeneration, and Diabetic Peripheral Neuropathy

**DOI:** 10.1111/jnc.70529

**Published:** 2026-08-04

**Authors:** Gentry Totta‐Griese, Douglas E. Wright

**Affiliations:** ^1^ Department of Anesthesiology The University of Kansas Medical Center Kansas Kansas USA

## Abstract

Methylglyoxal is a highly reactive by‐product of glycolysis that is elevated in diabetes and contributes to the development of diabetic peripheral neuropathy (DPN). DPN is characterized by nerve degeneration, typically manifesting in patients' extremities. This leads to patients experiencing numbness, burning, and pain. It has been established that elevated methylglyoxal levels lead to nociception, but the broader cellular effects of methylglyoxal on neurons in the dorsal root ganglia (DRG) remain poorly understood. This review provides mechanistic insight regarding methylglyoxal's impact on various cell types and disease contexts. Five main mechanisms were identified: protein glycation, proteostasis change, oxidative stress, metabolic changes, and increased inflammation. These mechanisms are thoroughly interconnected, contributing to cellular dysfunction associated with DPN. We propose that methylglyoxal functions as a central mediator in cellular stress, linking hyperglycemia and elevated glycolysis to neuronal dysfunction in DPN. There is extensive evidence that these mechanisms are methylglyoxal‐driven in other cell types and diseases, but a gap in the field remains in determining whether and how they occur in DRG neurons. This is particularly important, as DPN is a frequent comorbidity in diabetes and metabolic diseases and greatly affects patients' quality of life. Understanding the effect of methylglyoxal on DRG in relation to these mechanisms will provide novel insights into the development of DPN and lead to new therapeutic targets.

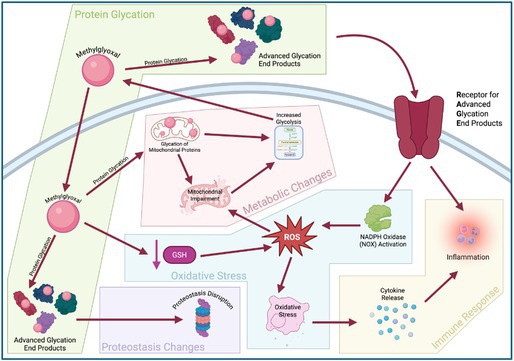

## Graphical abstract

This work reviews how the metabolic by‐product methylglyoxal may contribute to the development of diabetic peripheral neuropathy (DPN), a common complication of diabetes characterized by nerve damage and chronic pain in the extremities. In diabetes, methylglyoxal levels rise due to increased glucose metabolism and reduced detoxification capacity. While it is already known to trigger pain by increasing neuronal excitability, this review highlights its broader role in damaging peripheral sensory neurons in the dorsal root ganglia (DRG), which are key drivers of pain and sensory signaling in DPN. Across multiple biological systems, there is an observed pattern of methylglyoxal driving five main cellular stress mechanisms. Methylglyoxal increases protein glycation and formation of advanced glycation end products (AGEs), disrupts protein homeostasis (proteostasis), increases oxidative stress, contributes to mitochondrial dysfunction, and promotes inflammatory signaling. These processes reinforce one another, and this review suggests these mechanisms may also occur in DRG leading to neuronal injury, likely contributing to both axon degeneration and persistent neuropathic pain. The central message is that methylglyoxal is not just a pain‐inducing molecule, but a broader metabolic stress mediator that may link diabetes to progressive nerve degeneration. However, its specific effects within DRG neurons remain insufficiently studied, highlighting a key gap in understanding DPN and a potential direction for future therapeutic development.

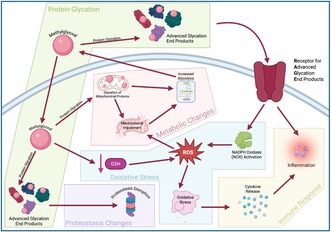

**Abbreviations**
AGE: advanced glycation end products
ATP: adenosine triphosphate
DHAP: dihydroxyacetone phosphate
DNA: deoxyribose nucleic acid
DPN: diabetic peripheral neuropathy
DRG: dorsal root ganglion
G3P: glyceraldehyde 3‐phosphate
GAPDH: glyceraldehyde 3‐phosphate dehydrogenase
GLO1: glyoxalase 1
GLO2: glyoxalase 2
GLUT1: glucose transporter 1
GLUT3: glucose transporter 3
IL‐#: interleukin #
LPS: lipopolysaccharide
NAD^+^
: nicotinamide adenine dinucleotide
NAV1.8: voltage‐gated sodium channel 1.8
NF‐kB: nuclear factor kappa‐light‐chain‐enhancer of activated B cells
NMN: nicotinamide mononucleotide
RAGE: receptor of advanced glycation end products
ROS: reactive oxygen species
SARM1: sterile alpha and Toll/interleukin‐1 receptor motif‐containing protein 1
TRP: transient receptor potential
TRPA1: transient receptor potential ankyrin 1
TRPV1: transient receptor potential vanilloid 1

## 1 Background

In 2025, it is reported that 1 in 9 adults is living with diabetes (International Diabetes Federation 2026). Diabetic peripheral neuropathy (DPN) is a comorbidity of diabetes that affects up to half of patients with diabetes and is characterized by degeneration of peripheral nerves, typically manifesting in the extremities (Mauermann and Staff NP 2026; Staehelin Jensen 2023; Slouma et al. 2024; Dave and Patel 2025). Patients experience numbness, tingling, and pain, which can result in reduced mobility and autonomy.

Despite the high prevalence and clinical burden, there are few effective treatment options, despite many failed attempts to identify new approaches (Table 1). Existing treatments are limited to symptom management, particularly mitigating pain and glycemic control. There have been multiple clinical trials for diabetes, diabetic peripheral neuropathy, and diabetic nephropathy that have failed. These treatments targeted mechanisms such as reducing oxidative stress, AGE cross‐linking, and microvascular complications, and increasing the survival of small nerve fibers (Table 1). However, glucose regulation is often ineffective in preventing neuropathy in type 2 diabetes. This highlights that hyperglycemia is not the sole contributor to DPN progression, underscoring the need to understand other factors that contribute to DPN and its associated pain.

**TABLE 1:** Highlight of clinical trials for diabetes therapeutics.

Intervention	Mechanism	Trial design	Main outcomes measured	Main result	Conclusion	Citations
Benfotiamine	Body converts to thiamine or vitamin B1. Seen to decrease glycemia and leptin concentrations.	BOND study: randomized, double‐blind, placebo‐controlled, 12 months; type 2 diabetes with mild‐moderate symptomatic DSPN; *n* = 57 randomized.	Corneal nerve fiber density, intraepidermal nerve fiber density, SOD2 area, motor nerve conduction velocity, Cold detection threshold, or secondary morphometric, functional, clinical, or QoL endpoints	No significant difference vs. placebo in primary and secondary outcomes.	Benfotiamine didn't improve outputs compared to placebo control.	(Barragan‐Iglesias et al. 2019)
Benfotiamine		24‐month randomized, double‐blind, placebo‐controlled trial in longstanding type 1 diabetes *n* = 59.	Peripheral nerve function assessed longitudinally.	No significant effect on nerve conduction velocity or inflammatory markers, although there was a significant increase in thiamine levels.	There was no increase in peripheral never function or inflammatory markers in patients from long‐term high‐dose benfotiamine.	(Yousuf et al. 2025)
Pyridoxine (vitamin B6)	Pyridoxine, also known as vitamin B6 functions as an antioxidant that suppresses both reactive oxygen species and advanced glycation end products.	This study was randomized control open label interventional study over 4 weeks, where participants were given either metformin (500 mg/day), Metformin (500 mg/day) with pyridoxine (300 mg/day), or a non‐pharmacological therapy. Participants were 30–65 years old with Type 2 diabetes and HbA1c less than or equal to 7.5%. *N* = 108.	Primary outcomes of fasting plasma glucose and glycated hemoglobin, and secondary outcomes of fasting plasma insulin, insulin resistance, vitamin B6 and indoleamine 2,3 dioxygenase levels.	All treatment groups saw a significant decrease over 4 weeks in the primary outcomes secondary outcomes of fasting plasma insulin and insulin resistance. Both metformin groups saw significant changes in vitamin B6 and indoleamine 2,3 dioxygenase levels.	Adding B6 to metformin treatment had a positive effect as diabetes contributes to a vitamin B6 deficiency.	(Enders et al. 2022)
Aminoguanidine (pimagedine)	Aminoguanidine prevents AGE facilitated cross‐linking of proteins in tissue.	ACTION II: A double‐blind, randomized, placebo‐controlled trial comparing a placebo to two different dose of aminoguanidine on the progression of nephropathy in type 2 diabetic patients. Participants were between 30 and 70 years of age with a diabetes diagnosis for atleast a year. *N* = 599.	Progression of diabetic nephropathy.	The trial was ended early due to safety concerns and the benefit of aminoguanidine on nephropathy progression being tenuous.	Important question for the pathway overall, but not a neuropathy‐specific efficacy trial. It likely helps explain why this mechanistically attractive target did not mature into successful DPN therapy.	(Liu et al. 2017; Griggs et al. 2017)
Tolrestat	Is an inhibitor of aldose reductase, which is in the polyol pathway and in hyperglycemic conditions converts glucose to sorbitol leading to oxidative stress.	A double‐blinded, placebo controlled trial for 52 weeks. *N* = 372.	Motor nerve conduction velocity (MNCV).	Patients in placebo group had significantly reduced MNCV. With‐drawal from long term tolrestat treatment led to a harmful impact on DPN outcomes.	Polyol pathway may be an effective target for DPN treatment. Studies discontinued in 1997 due to severe liver toxicity and death.	(Mian et al. 2025; Sima et al. 1993)
Recombinant human NGF	As a neurotrophic factor, NGF helps the survival of small fiber sensory neurons.	12‐month, Randomized, double‐blind, placebo‐controlled phase 3 trial in males and females from 18 to 74 years old. *N* = 1019.	Neuropathy Impairment Score at baseline, 12, 24, and 48 weeks.	There was not improvement in the Neuropathy Impairment Score or Quantitative Sensory Testing in the treatment group.	Phase 3 trial failed due to no observed benefit from treatment.	(Tolrestat 2005)
α‐lipoic acid	α‐lipoic acid shows functions as a reducer of oxidative stress.	NATHAN 1 Trial: randomized double‐blind parallel‐group trial in patients with mild to moderate distal symmetric sensorimotor polyneuropathy. Patients were either given 600mg of α‐lipoic acid once daily or a placebo for 4 years. *N* = 460.	Neuropathy impairment score.	From baseline to end of trial there were not any significant differences between groups.	There was not a significant decline in the end point for the placebo group, indicating that the treatment was not beneficial.	(Bonhof et al. 2022)
Engensis (VM202)	DNA plasmid that encodes for hepatocyte growth factor which has previously been seen in phase II to alleviate pain in DPN.	A two‐part, double‐blind, randomized, parallel design, placebo‐controlled phase III trial. The first part is 9‐montsh (*N* = 500) and then an extension in a subset of patients (*N* = 101) to 12 months. Treatment was given to patients in the calf muscles on Days 0, 14, 90, and 104.	Numerical rating scale to record a pain score.	In the first section of the trial, VM202 didn't meet any endpoints. However, in the second section there was pain relief between VM202 and placebo groups.	Due to the differences between the two sections of the studies, there is need for an additional trial.	(Fraser et al. 2012)
C‐Peptide	Decrease of C‐peptide seen in diabetes that may contribute to micorvascular complications.	Phase 2 trial conducted on patients with diabetes and peripheral neuropathy either received a weekly low dose or high dose of C‐peptide or a placebo for 52 weeks. *N* = 250.	Sural nerve conduction velocity (SNCV) and vibration perception threshold (VPT) at Weeks 0, 26, and 52.	C‐peptide did not improve SNCV but did improve VCT.	Did not have robust efficacy.	(College A‐RU 2023)
Trans‐resveratrol and hesperetin	Induces expression of Glyoxalase‐1	This study was conducted over 8‐weeks and was double‐blinded, randomized, placebo‐controlled crossover study. 29 subjects with 9 participants having pre‐diabetes and all 29 participants with impaired metabolic health. Participants took a oral capsule prior to breakfast daily of either placebo or tRES‐HESP (90 mg tRES, 120 mg HESP).	Insulin sensitivity via oral glucose insulin sensitivity (OGIS) index in oral glucose tolerance tests (GTTs).	Reduced insulin resistance and decreased dysglycemia, blood pressure, vascular inflammation, dyslipidemia, and plasma methylglyoxal.	Modulating glyoxalase 1 and reducing methylglyoxal had a positive impact on metabolic impairment.	(Xue et al. 2025)
Combinations of amitriptyline, pregabalin, and duloxetine	Testing combinations of leading diabetic peripheral neuropathic pain therapies: amitriptyline, pregabalin, and duloxetine, which act by enhancing serotonin/norepinephrine signaling or reducing neurotransmitter release.	Multicenter, double‐blind, randomized, crossover trial in patients with diabetic peripheral neuropathic pain at a daily pain numeral rating scale (NRS) of 4 or higher using 130 participants over the 16 week trial.	Primary outcome was difference in daily NRS averaged over 7‐days.	In every treatment plan, the averaged NRS score decreased from baseline to the 16‐week timepoint.	All treatment pathways and monotherapies had similar analgesic efficacy.	(Tesfaye et al. 2022)
High frequency spinal cord stimulation (HF‐SCS)	Spinal cord stimulation has been used as a nonpharmacological pain relief approach.	Randomized, 6‐month trial with 216 particpants. Conventional medical management (CMM) used as control group.	Primary outcome was percentage of participants with 50% or more pain relief using visual analogue scale (VAS) compared to baseline at 3 months and 6 months.	79% of the HF‐SCS group saw improved VAS scores compared to 5% in the CMM group.	Potential nonpharmacological approach for painful diabetic peripheral neuropathy.	(Petersen et al. 2021)
Capsaicin	Activates and desensitizes TRPV1‐expressing nociceptive nociceptors leading to the pain reduction.	Randomized, double‐blind, placebo‐controlled 12‐week trial of 369 adults with painful diabetic peripheral neuropathy (PDPN). Patients received a single 30‐min application of either capsaicin 8% patch (*n* = 186) or placebo patch (*n* = 183) to painful areas of the feet.	Primary outcome: Percent reduction in average daily pain score from baseline to Weeks 2–8. Secondary outcomes included time to treatment response, sleep interference, safety, and sensory function.	Capsaicin 8% patch produced a greater reduction in pain than placebo by week 2 and slight improvements in sleep quality. Adverse events were similar between groups except for application‐site reactions, and no sensory deterioration was observed.	A single 30‐min application of capsaicin 8% patch provided modest but significant pain relief and improved sleep in PDPN patients, with minimal systemic side effects and no evidence of worsening sensory function.	(Simpson et al. 2017)
SGLT2 inhibitor (empagliflozin)	Promotes increased urinary excretion of glucose, improving glucose metabolism and reducing glucotoxicity.	20 week study that included 50 participants with diabetic neuropathy randomly assigned to either control or treatment group. Treatment group given 10 mg of empagliflozin daily.	Outcomes of fasting blood glucose, hemoglobin A1c, serum creatinine levels and nerve conduction velocity measured for baseline and at the 20‐week timepoint.	Significant reduction in fasting blood glucose, hemoglobin A1c, and serum creatinine levels and improved nerve conduction velocity in the treatment group at week 20 compared to baseline.	Had benefits for DPN symptoms and could be a potential therapeutic option.	(Vafaeinasab et al. 2025)

*Note:* This table highlights prominent clinical trials aimed at mitigating diabetes related complications, including DPN, DPN related pain, and metabolic changes. These trials had mixed results, with many of the trials aimed at alleviating pain and metabolic outcomes having successful findings, while trials geared towards neuropathy and nerve health did not see robust benefit (Sima et al. 1993; Tolrestat 2005; Bonhof et al. 2022; Fraser et al. 2012; College A‐RU 2023; Thornalley 2003; Freedman et al. 1999; Santiago et al. 1993; Apfel et al. 2000; Ziegler et al. 2011; Kessler et al. 2021; Wahren et al. 2016; Tesfaye et al. 2022; Petersen et al. 2021; Xue et al. 2025; Vafaeinasab et al. 2025; Simpson et al. 2017).

Dorsal root ganglia (DRG) are essential to DPN pathology, as they house the cell bodies of peripheral sensory nerves, which are typically affected by DPN and related neuropathic pain (Bhandari et al. 2021; Doty et al. 2022). Sensory neurons in the DRG are particularly vulnerable to metabolic stress due to their preference for glucose and their long axonal projections, which require substantial energy for maintenance (Miyashita et al. 2023). Interestingly, DRG neurons express GLUT1 and GLUT3, both insulin‐independent glucose transporters, thereby permitting intracellular glucose accumulation even in insulin‐resistant contexts (Rawat and Morrison 2021). This positions DRG neurons as especially susceptible to hyperglycemia‐related stressors.

**FIGURE 1:**
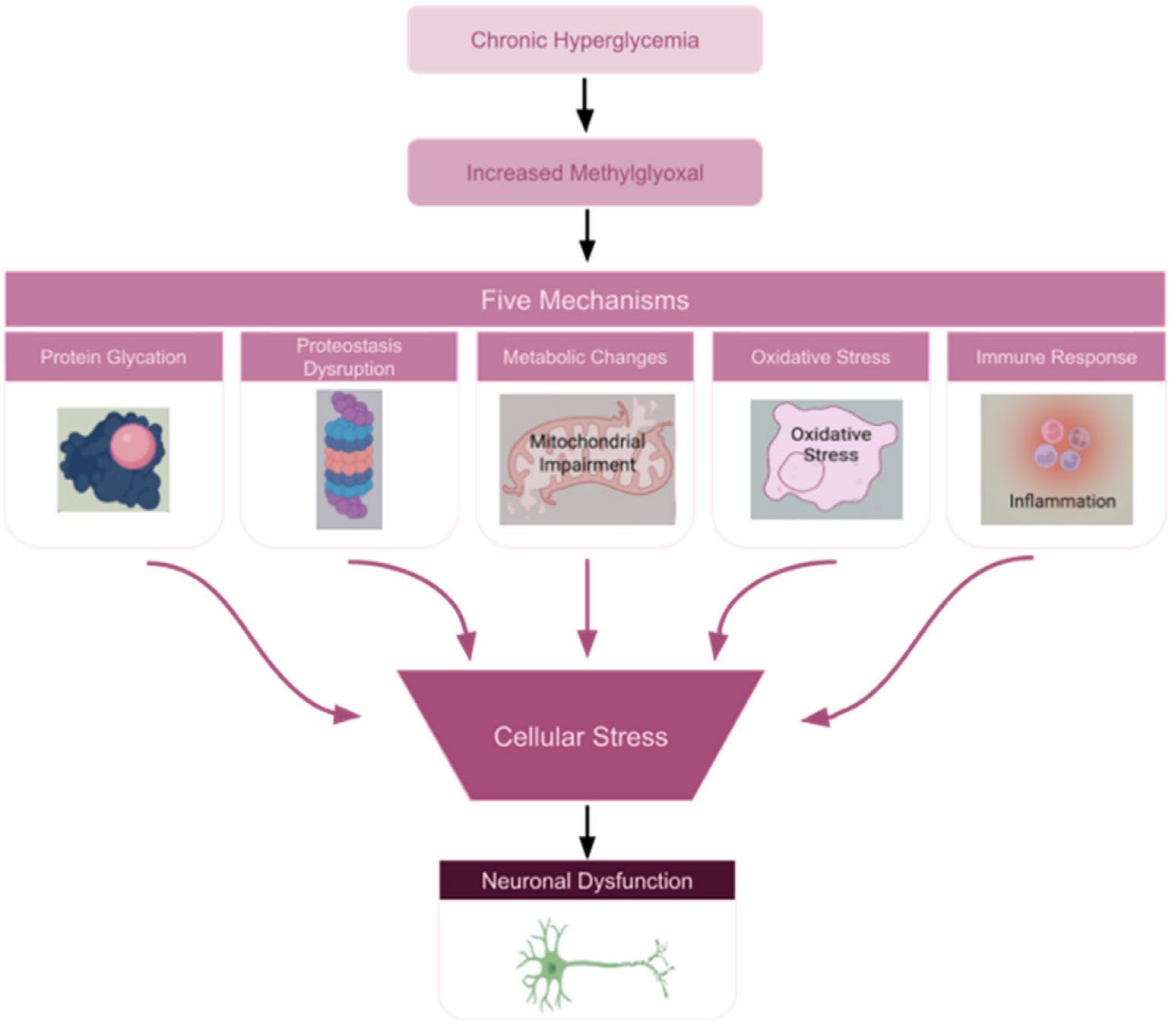
Five proposed mechanisms underlying neuronal dysfunction. Schematic illustration of possible mechanisms that contribute to cellular stress and neuronal dysfunction.

Among these stresses, methylglyoxal has been identified as a key candidate to link altered glucose metabolism to neuronal dysfunction (Griggs et al. 2021; Huang et al. 2012). Methylglyoxal is a by‐product of glycolysis that is made at physiological levels and elevated in diabetes (Allaman et al. 2015; Beisswenger 2014; Coccini et al. 2023; Bierhaus et al. 2012). It is a highly reactive glycation agent leading to the formation of advanced glycation end products (AGEs) (Schalkwijk and Stehouwer 2020; Price and Knight 2007; Ramasamy et al. 2006; Maessen et al. 2015). Under physiological conditions, methylglyoxal is scavenged by the glyoxalase system (Maessen et al. 2015; Thornalley 2008; Stratmann et al. 2017). However, in hyperglycemic conditions, methylglyoxal levels increase while glyoxalase activity diminishes, leading to methylglyoxal levels exceeding the glyoxalase's capacity (Stratmann et al. 2017; Cianfruglia et al. 2020; Kim et al. 2020; Queisser et al. 2010). Increased methylglyoxal levels have been strongly associated with diabetic complications, including DPN (Beisswenger 2014; Bierhaus et al. 2012; Schalkwijk and Stehouwer 2020; Maessen et al. 2015; Nigro et al. 2017) (Figure 1).

The relationship between methylglyoxal and neuropathy has been extensively explored in the context of nociception. Methylglyoxal modifies transient receptor potential ankyrin 1 (TRPA1), leading to TRPA1 activation (Andersson et al. 2013; Eberhardt et al. 2012). Through post‐translational modifications, methylglyoxal modifies the voltage‐gated sodium channel NAV1.8, increasing neuronal excitability and leading to nociception (Bierhaus et al. 2012; Andersson et al. 2013; Eberhardt et al. 2012; Huang et al. 2016; Ueno et al. 2024). Additionally, work has shown that methylglyoxal leads to Nav1.7‐mediated hyperalgesia through increased expression of the channel (Cheng et al. 2019). Methylglyoxal has also been shown to sensitize sensory neurons in DPN, inducing neuropathic pain by activating the integrated stress response (Mian et al. 2025; Barragan‐Iglesias et al. 2019; Yousuf et al. 2025). Previous research has utilized various experimental paradigms demonstrating the noxious impact of methylglyoxal in both rats and mice through assessing itch, mechanical threshold, thermal latency, and nociceptive behavior (Huang et al. 2016; Cheng et al. 2019; Yousuf et al. 2025; Enders et al. 2022; Liu et al. 2017; Griggs et al. 2017). While these results solidify methylglyoxal as a causative agent of pain, they do not fully explain its role in axon degeneration and broader neuronal dysfunction within DRG in DPN. Understanding these mechanisms is crucial to the development of novel therapeutics.

## 2 Methylglyoxal: Sources, Metabolism, and Detoxification

### 2.1 Production of Methylglyoxal

Methylglyoxal is a by‐product of glycolysis that is endogenously produced at physiological levels. The rate of methylglyoxal formation varies across tissues and cells and is influenced by cellular metabolism and physiological conditions. Because it is formed by glycolysis, it is produced in almost every cell. Methylglyoxal is formed from the intermediates dihydroxyacetone phosphate (DHAP) and glyceraldehyde 3‐phosphate (G3P), which can spontaneously degrade to methylglyoxal (Allaman et al. 2015; Murata‐Kamiya and Kamiya 2001). Methylglyoxal can be formed through additional pathways, including lipid peroxidation, DNA degradation, and aminoacetone oxidation.

Exogenous sources can also contribute to methylglyoxal accumulation. In particular, methylglyoxal is abundant in lipid‐rich foods. The most prominent way it is produced in foods is through the Maillard reaction, which causes meats and breads to brown and form a crust. Additionally, animal products high in protein and fat are also methylglyoxal‐rich. Thus, dietary choices can contribute to methylglyoxal accumulation (IARC 1991; Li et al. 2024; Uribarri et al. 2010; Tan et al. 2008).

Methylglyoxal is incredibly reactive and has a short half‐life, making it difficult to measure the levels. It is estimated that free methylglyoxal ranges from 1 to 5 μM intracellularly (Jun and Kool 2020). However, due to its short half‐life, the location and time of formation can lead to significantly higher local concentrations. It has been shown that 90%–99% of cellular methylglyoxal is bound. It is predicted that the total cellular amount of methylglyoxal, which includes both free and bound, can be up to 300 μM (Allaman et al. 2015; Rabbani and Thornalley 2014a). Methylglyoxal is a ubiquitous by‐product of glycolysis whose production varies with tissue type and metabolic state, reflecting cellular metabolism. Its accumulation arises from multiple endogenous pathways and dietary sources, linking it to broader metabolic and oxidative stress. Although free levels are low, its high reactivity, protein binding, and localized spikes suggest that total methylglyoxal burden drives its biological impact.

### 2.2 Detoxification Pathways

The primary route for methylglyoxal detoxification in the cell is via the glyoxalase pathway. The glyoxalase pathway uses two enzymes, glyoxalase 1 (GLO1) and glyoxalase 2 (GLO2), with GLO1 being the rate‐limiting enzyme. Methylglyoxal combines with glutathione to form hemithioacetal. Then, in the first enzyme‐mediated reaction, GLO1 converts hemithioacetal into S‐lactoylglutathione. GLO2 makes S‐lactoylglutathione into D‐lactate while recycling glutathione.

There are additional ways methylglyoxal is degraded in the cell. Glyoxalase III, which is also known as DJ‐1 or PARK7, is an enzyme that can degrade methylglyoxal into D‐lactate without glutathione (Jun and Kool 2020; Choi et al. 2023; Yumnam et al. 2020). In instances of reduced GLO1, compensatory mechanisms involving aldose reductase and aldehyde dehydrogenase (ALDH) can help detoxify methylglyoxal. Aldose reductase is more efficient than ALDH at breaking down methylglyoxal due to its higher affinity for methylglyoxal. Aldose reductase is associated with diabetic complications. It can convert methylglyoxal into acetol through a glutathione‐dependent pathway or can form propanediol through a glutathione‐independent pathway. Additionally, ALDH can convert methylglyoxal into pyruvate via an NAD‐dependent mechanism (Lai et al. 2024). Thus, methylglyoxal formation and breakdown are highly regulated and robust processes, underscoring the importance of maintaining low levels.

### 2.3 Factors Elevating Methylglyoxal in Diabetes

There are multiple factors in diabetes that contribute to the elevated levels of methylglyoxal. First, methylglyoxal is a conventional by‐product of glycolysis, and increasing glycolysis increases methylglyoxal levels, as seen in diabetes. In diabetes, hyperglycemia increases methylglyoxal levels (Silhavy et al. 2020). Second, GLO1 levels are well characterized as decreased in diabetes (Rabbani and Thornalley 2014b). Because GLO1 is the rate‐limiting enzyme in the glyoxalase system, its downregulation in diabetes reduces glyoxalase pathway activity and limits methylglyoxal detoxification (Silhavy et al. 2020).

Third, cellular levels of reactive oxygen species (ROS) are increased in diabetes. An increase in ROS levels can decrease glutathione levels. It is well documented that glutathione levels decrease during diabetes (Sekhar et al. 2011; Darmaun et al. 2005). Glutathione is a vital cofactor for the glyoxalase cycle. Decreases in glutathione can reduce the efficiency of the glyoxalase cycle, thereby contributing to methylglyoxal accumulation (Rabbani et al. 2018; Masterjohn et al. 2013). Together, diabetes promotes methylglyoxal accumulation through hyperglycemia‐driven increased production and impaired detoxification due to reduced glyoxalase‐1 activity and glutathione depletion.

## 3 Key Mechanisms Underlying Methylglyoxal Cellular Impact

### 3.1 Protein Glycation

Methylglyoxal is a strong glycating agent that modifies lipids and proteins, creating AGEs (Beisswenger 2014). Methylglyoxal is a small, polar molecule. As a dicarbonyl, methylglyoxal is highly reactive (Allaman et al. 2015; Schalkwijk and Stehouwer 2020; Ramasamy et al. 2006; Nigro et al. 2017). Through non‐enzymatic reactions, it binds to amino groups, leading to irreversible modification (Schalkwijk and Stehouwer 2020; Rabbani and Thornalley 2014b; Lai et al. 2022). Although methylglyoxal has a short half‐life, AGEs are stable and can remain in the body for months. AGEs can have detrimental impacts through crosslinking to structural and basement membranes (Semba et al. 2010). or even years (Fournet et al. 2018). Consequently, AGEs that disrupt cellular structures affect permeability and cell motility (Allaman et al. 2015).

Methylglyoxal glycates cellular molecules to form AGEs (Allaman et al. 2015; Schalkwijk and Stehouwer 2020; Ramasamy et al. 2006; Lai et al. 2022), and glycation can affect metabolic enzymes, mitochondrial proteins, transcriptional regulators, cytoskeletal components, and extracellular matrix proteins. Glycation impairs protein–protein interactions, modifies catalytic activity, and decreases protein stability. This effect contributes to the mechanistic basis of stress responses in cells observed across various diseases (Rabbani and Thornalley 2014a; Fournet et al. 2018; Zgutka et al. 2023; Ott et al. 2014).

Additionally, AGEs can activate the receptor for advanced glycation end products (RAGE). RAGE is involved in numerous cell signaling pathways. Through RAGE activation, AGEs can upregulate inflammatory molecules (Ramasamy et al. 2006; Vlassara and Uribarri 2014). Methylglyoxal can also cause nucleic acid damage and chromosomal instability. By modifying deoxyguanosine residues, methylglyoxal can induce breaks in double‐stranded DNA (Donnellan et al. 2025).

The relationship between methylglyoxal, AGE formation, and RAGE activation is an important mechanism contributing to various diseases. Research has aimed to inhibit RAGE as a potential therapeutic option. Azileragon is a RAGE inhibitor that prevents STZ‐induced DPN and mechanical hypersensitivity in mice. Additionally, Azileragon is expected to reduce cognitive impairment in mild Alzheimer's Disease cases (Reddy et al. 2023). Additional work has demonstrated the role of RAGE in proinflammatory macrophage infiltration in DPN, leading to insulin sensitivity and reduced retrograde axonal transport in the sciatic nerve, both of which were protected against by bone marrow transplants from RAGE‐null mice (Osonoi et al. 2022). This research further supports the clinical application of understanding and targeting methylglyoxal‐mediated AGE formation mechanisms that contribute to DPN.

Methylglyoxal‐induced protein glycation has also been implicated in cancer. Diabetes increases cancer risk because higher fasting glucose levels correlate with increased risk for cancer and cancer mortality (Giovannucci et al. 2010; Chiavarina et al. 2014). Interestingly, methylglyoxal acts as both a promoter and an inhibitor of tumorigenesis depending on the concentration (Rabbani et al. 2018; Wang et al. 2025; Kim et al. 2022; Kong et al. 2024).

Shifting focus to specific cancers, diabetes increases breast cancer mortality, while metformin has been shown to lower cancer risk (Chiavarina et al. 2014; Parsons et al. 2026; Saraei et al. 2019). Interestingly, there is a lower amount of methylglyoxal‐derived AGEs in triple‐negative breast cancers (Chiavarina et al. 2014). In melanoma, AGEs stimulated tumor growth and migration, and RAGE inhibition was protective against tumor growth and metastasis in mice (Nakamura et al. 2017). In pancreatic cancer, RAGE activation promoted autophagy and the development of pancreatic tumors (Kang et al. 2012). In lung cancer, AGEs were identified in tumors, and in some patients, higher AGE fluorescence was associated with better survival (Bartling et al. 2011). Advanced glycation end product generation of AGEs and RAGE activation can activate various transcription factors, resulting in tumor‐promoting inflammation (Azizian‐Farsani et al. 2020). AGEs and RAGE activation are also associated with activation of carbohydrate response element‐binding protein (ChREBP), leading to increased glycolysis and anabolic metabolism that support the proliferation of cancer cells, resulting in a Warburg‐like metabolic shift (Chen et al. 2017).

From the current research, its clear that methylglyoxal plays a complex role in cancer: AGE formation promotes tumorigenesis via RAGE, but methylglyoxal is also toxic to cancer cells, leading to apoptosis (Rabbani et al. 2018; Kim et al. 2022; Kong et al. 2024). Ultimately, methylglyoxal appears to be a contributing factor in cancer progression through oxidative stress, inflammation, migration, and metastasis, and metabolic reprogramming (Kong et al. 2024; Yamagishi et al. 2015).

There is already a close relationship between diabetes and cardiovascular disease, with methylglyoxal playing a role in both pathologies. Within cardiovascular disease, AGEs can lead to atherosclerosis and plaque rupture, which is a main contributor to cardiovascular disease‐related death (Hanssen et al. 2014). Additionally, methylglyoxal can lead to endothelial cell damage and vascular dysfunction, both of which are risk factors for cardiovascular disease (Price and Knight 2007; Vulesevic et al. 2014).

Ultimately, methylglyoxal, which promotes protein glycation and leads to the accumulation of AGEs, forms the foundation on which many of the mechanisms addressed below are built. Methylglyoxal‐induced protein glycation is interconnected with metabolic changes, oxidative stress, inflammation, and proteostasis, as it drives these mechanisms through glycation.

### 3.2 Proteostasis Disruption

Methylglyoxal's role as a potent glycating agent can lead to protein misfolding, activation of the unfolded protein response signaling pathway, proteasomal overload, apoptosis, cellular senescence, and dysregulation of various cell signaling pathways (Queisser et al. 2010). A subset of studies has identified a pattern of proteostasis stress caused by methylglyoxal‐induced protein damage. In SH‐SY5Y human neuroblastoma cells, methylglyoxal induced significant changes in protein synthesis and cellular structural integrity, leading to endoplasmic reticulum stress (Wang, Boeren, et al. 2024). It is also known that methylglyoxal can cause toxic DNA‐protein crosslinks. Their interactions can prevent DNA replication, transcription, and repair. DNA‐protein crosslinks can also affect protein expression and function. Human embryonic kidney cells (HEK293T) were treated with 50 *μ*M–10 mM methylglyoxal for 2, 4, or 24 h, resulting in 256 proteins identified as occurring in methylglyoxal‐induced DNA‐protein cross‐linking (Hurben et al. 2024). It has also been observed that this cross‐linking results in the accumulation of damaged proteins. Methylglyoxal also impairs the ubiquitin‐proteasome system (Bento et al. 2010), and the accumulation of AGEs can inhibit the function of the ubiquitin‐proteasome system (Queisser et al. 2010; Ott et al. 2014). These studies highlight the pattern of proteostasis disruption acting as an intermediate connecting carbonyl stress to cellular dysfunction.

### 3.3 Oxidative Stress

Methylglyoxal also plays a key role in the accumulation of ROS. Methylglyoxal treatment led to oxidative stress, accumulation of ROS, and decreased levels of antioxidants and ROS‐scavenging proteins (Coccini et al. 2023). This has been reported in a variety of tissues and cell types, including human umbilical vein endothelial cells (HUVEC), human endothelial cells, H9c2 cardiomyoblasts, rat INS‐1 pancreatic beta cells, the intestinal cell line CaCo‐2, brain tissue from humans and mice, the kidney epithelial cell line HK‐1, HepG2 cells, and the human endothelial cell line EA.hy926. Although methylglyoxal treatment increased ROS across cell types, the proposed mechanisms varied (Seo et al. 2014; Chen et al. 2022; Suantawee et al. 2020; Wang et al. 2022; Wang, Chen, et al. 2024).

Methylglyoxal also contributes to oxidative stress by generating AGEs that activate RAGE (Wautier et al. 2001). Through RAGE signaling, NADPH oxidase is activated, leading to nitric oxide (NOX) production (Seryogina et al. 2024; Chen et al. 2018). NOX is a type of ROS, and ROS formation can activate the transcription factors NF‐κB and AP‐1, leading to inflammation through the release of chemokines and proinflammatory cytokines (Wang, Chen, et al. 2024; Kim et al. 2021).

As discussed above, the glyoxalase system is the primary pathway for methylglyoxal degradation. In the glyoxalase system, glutathione, a prominent antioxidant, is used as a cofactor. Although glutathione isn't consumed, increases in methylglyoxal concentration require more glutathione; this could result in less available glutathione to be used as an antioxidant (de Bari et al. 2020). Additionally, methylglyoxal was found to decrease glutathione levels and activity in both in vitro and in vivo settings. In vitro, methylglyoxal decreases glutathione levels in HUVECs, HepG2 cells, and INS‐1 pancreatic beta cells from rats (Seo et al. 2014; Suantawee et al. 2020; Wang et al. 2022; Wang, Chen, et al. 2024; Deng et al. 2024). In vivo, glutathione was decreased in male C57BL/6 mice (Wang et al. 2022; Wang, Chen, et al. 2024; Deng et al. 2024).

The methylglyoxal‐induced accumulation of ROS was attenuated by antioxidant treatment (Deng et al. 2024; Jarisarapurin et al. 2021). In human brain microvascular endothelial cells, methylglyoxal increased ROS release, which was reversed by aminoguanidine and N‐acetylcysteine (Chen et al. 2022; Deng et al. 2024; Jarisarapurin et al. 2021).

Methylglyoxal has been consistently shown to induce oxidative stress and promote ROS accumulation across multiple experimental models. Elevated ROS levels are a hallmark of cellular stress and contribute to the pathogenesis of numerous diseases. Determining whether methylglyoxal drives ROS accumulation in peripheral nerve tissue will provide important insight into the cellular stress mechanisms underlying diabetes and DPN.

### 3.4 Mitochondrial Impairment

Several recent studies have reported oxidative stress from methylglyoxal exposure, mediated by a variety of pathways. ROS production associated with methylglyoxal glycation disrupts glutathione metabolism and the activity of antioxidants (Wang, Chen, et al. 2024; Deng et al. 2024; Liccardo et al. 2024). Multiple studies have postulated that oxidative stress contributes to mitochondrial dysfunction, as evidenced by reduced ATP production, increased mitochondrial ROS generation, changes in mitochondrial membrane potential, and impaired activity of electron transport complex proteins (Bhatti et al. 2017; Shibanuma et al. 2011). Additionally, methylglyoxal can glycate mitochondrial proteins, leading to mitochondrial impairment, ROS production, and oxidative stress (Stratmann et al. 2017). These changes contributed to metabolic impairment and cellular injury.

A common effect across multiple cell types was disruption of mitochondrial membrane potential (MMP). MMP is a measurement of the electrical gradient derived from the electron transport chain, which pumps electrons across the inner mitochondrial membrane. Measuring changes in MMP indicates mitochondrial disruption. A decrease in MMP after methylglyoxal treatment was seen in HUVEC, human endothelial cells, H9c2 cardiomyoblasts, and rat INS‐1 pancreatic beta cells (Wang, Chen, et al. 2024; Liccardo et al. 2024; Nuamnaichati et al. 2020; Yoo et al. 2020).

In SH‐SY5Y human neuroblastoma cells, a proteomic and metabolomic study revealed that methylglyoxal caused significant alterations in multiple metabolic pathways, including the mitochondria, the tricarboxylic acid cycle, cysteine and methionine metabolism, and arginine biosynthesis (Wang, Boeren, et al. 2024).

In patients with type 2 diabetes and DPN, magnesium levels were decreased and were inversely related to methylglyoxal levels. In DRG, one group observed that diminished mitochondrial activity, together with triosephosphate supplementation, which generates endogenous methylglyoxal, led to neurite degeneration. The authors suggested that magnesium supplementation was protective and concluded that axon degeneration is associated with impaired energy metabolism and magnesium deficiency (Strom et al. 2021).

Treatment with N‐acetyl cysteine, a precursor to glutathione, reversed methylglyoxal‐mediated mitochondrial damage and mitophagy. This study proposed that oxidative stress induced by increased methylglyoxal, as seen in diabetes, contributes to mitochondrial damage and mitophagy (Kim et al. 2020). This points to either the effect of scavenging methylglyoxal alleviating mitochondrial damage, or to ROS accumulation resulting from methylglyoxal accumulation causing mitochondrial damage.

Exercise is often tested as an intervention for disease prevention due to its many positive effects. In preclinical studies, methylglyoxal was administered to mice undergoing endurance training. Methylglyoxal causes exercise resistance, meaning it prevents mitochondrial adaptations, activation of insulin signaling, and upregulation of proteins involved in mitochondrial biogenesis in fast‐twitch muscles, as seen in the exercise group (Egawa et al. 2022). Studies indicate that methylglyoxal causes detrimental metabolic changes, but this study also shows that it can prevent beneficial metabolic changes. Exercise is often used as an intervention for diabetes and DPN due to its positive effects on insulin resistance and blood glucose control. Understanding the role of methylglyoxal in metabolism and its relationship with exercise is important for the development of treatments for diabetes and DPN.

Although methylglyoxal is a by‐product of glycolysis, some studies report that its formation can increase glycolytic activity, thereby contributing to inflammation and metabolic impairment (Yang et al. 2022). One study using in vitro approaches and preclinical mouse models found that hyperglycemia resulted in decreased GLO1 (Rabbani and Thornalley 2014b). This effect on GLO1 led to increased methylglyoxal, thereby perpetuating a cycle of hyperglycemia, elevated methylglyoxal, and reduced GLO1 (Rabbani and Thornalley 2014b; Rabbani et al. 2018; Cortizo et al. 2022; Rabbani and Thornalley 2019). The glycolytic enzyme glyceraldehyde‐3‐phosphate dehydrogenase (GAPDH) is inhibited by methylglyoxal in mammalian neural stem cells, indicating another mechanism by which methylglyoxal can alter metabolic pathways (Rodrigues et al. 2020).

As previously discussed, methylglyoxal accumulation is associated with dysregulation of various metabolic pathways. Specically in cancer this includes impaired insulin signaling, altered glucose metabolism, and metabolic reprogramming. Methylglyoxal's glycation results in transcription factors and intermediates in signaling pathways being modified, resulting in metabolic flux. For example, methylglyoxal can impair the tumor suppressor BRCA2, inducing cancer‐associated DNA damage. This metabolic mechanism suggests that elevated methylglyoxal levels may bypass Knudson's two‐hit requirement, linking metabolic stress or increased glycolysis to the accumulation of mutations during cancer development (Kong et al. 2024).

Recent work has shown that elevated methylglyoxal levels can alter axon morphology and structure (Griggs et al. 2021; Coccini et al. 2023; Yousuf et al. 2025; Griggs et al. 2018). The mechanisms underlying axon degeneration are still being explored, but it has been suggested that these metabolic changes are key features leading to axon degeneration and loss (Baltan et al. 2010; Beirowski 2022; Harun‐Or‐Rashid et al. 2018). Additionally, new research has highlighted the relationship between mitochondria and SARM1 (Murata et al. 2013). SARM1, an enzyme involved in Wallerian degeneration, can be activated by metabolic changes and, once activated, contributes to mitochondrial changes that are likely upstream of, and directly related to, subsequent axon degeneration (Harun‐Or‐Rashid et al. 2018; Figley et al. 2021; Loreto et al. 2025; Sato‐Yamada et al. 2022). Many other neurodegenerative diseases, including Alzheimer's and Parkinson's, are also tightly coupled to metabolic changes (Griffith et al. 2008; Stinson et al. 2025). This highlights a key need for future research into whether the methylglyoxal‐related changes in axon morphology have underlying metabolic mechanisms. Taken together, this information indicates that methylglyoxal not only acts as a toxic carbonyl species but also powerfully regulates metabolic signaling pathways under both cellular and systemic stress conditions.

### 3.5 Inflammatory and Immune Signaling Activation

Numerous studies have examined changes in inflammatory signaling pathways following methylglyoxal exposure (Kim et al. 2021; Prantner et al. 2021; Prantner and Vogel 2025; Medeiros et al. 2021). Oxidative stress led to increased cytokine expression, immune cell activation, and inflammatory transcriptional responses associated with inflammation (Wang, Chen, et al. 2024; Medeiros et al. 2021). The immune and inflammatory responses were observed across various disease contexts, including cardiovascular disease, neurodegeneration, cancer, and metabolic disease (Wang et al. 2025; Medeiros et al. 2021; Vulesevic et al. 2016; Berdowska et al. 2023; Lin et al. 2018; Willemen et al. 2023). This highlights immune response as a prominent downstream effect of methylglyoxal‐linked pathology.

A clinical study has reported that methylglyoxal is upregulated in patients with sepsis (Prantner et al. 2021). Similarly, LPS‐treated mice had increased levels of macrophage‐produced methylglyoxal (Prantner et al. 2021; Prantner and Vogel 2025). In a different preclinical LPS model, LPS was used to induce acute lung injury in mice. Mice were then treated with methylglyoxal, which increased neutrophil infiltration in the airways and mRNA expression of IL‐1β and TNF‐α (Medeiros et al. 2021). In a different preclinical LPS model, LPS was used to induce acute lung injury in mice. Mice were then treated with methylglyoxal, which increased neutrophil infiltration in the airways and mRNA expression of IL‐1β and TNF‐α (Medeiros et al. 2021). In a rectal cancer model, methylglyoxal released by gut microbiota facilitated ROS‐induced ER stress, leading to immunogenic cell death and CD8+ T cells in the tumor microenvironment (Zhou et al. 2023). In a cardiovascular disease model, mice given methylglyoxal had significantly higher circulating levels of neutrophils, T‐cells, and CD68+ macrophages, indicating that increased methylglyoxal levels can contribute to inflammation (Hanssen et al. 2023).

**FIGURE 2:**
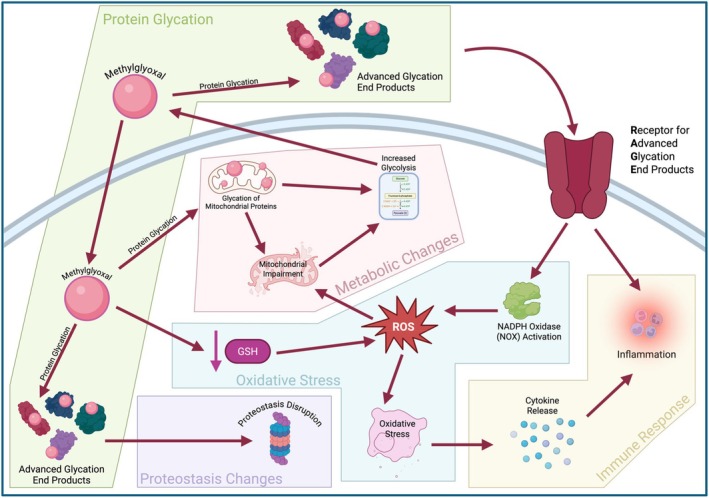
Five central mechanisms of methylglyoxal in cellular stress. Diagram demonstrating the five central mechanisms: Protein glycation, proteostasis changes, oxidative stress, metabolic changes, and immune response, and how they may be interconnected. Methylglyoxal is a potent glycating agent that binds to proteins, forming AGEs (protein glycation). As a glycating molecule, methylglyoxal can also modify mitochondrial proteins (metabolic changes). The formed AGEs bind to RAGE, leading to alterations in cellular pathways (proestasis changes) that contribute to inflammation (immune response) or to NOX activation. NOX activation promotes ROS accumulation (oxidative stress), which can lead to cytokine release (immune response) or mitochondrial impairment (metabolic changes). This diagram illustrates the interconnectedness of these mechanisms, highlighting methylglyoxal as a central mediator of cellular stress. We propose that the convergence of these mechanisms within DRG neurons contributes to axon degeneration and nociceptor sensitization in DPN.

In C57/BL6 male mice, methylglyoxal increased the pro‐inflammatory cytokines IL‐6 and IL‐1β and decreased the anti‐inflammatory cytokine IL‐10, providing strong in vivo evidence for a role of methylglyoxal in inflammation (Wang et al. 2022; Wang, Chen, et al. 2024). Collectively, these findings demonstrate that elevated methylglyoxal promotes inflammatory and immune activation across diverse disease models, likely through mechanisms involving ROS generation and cellular stress signaling. These observations, taken together, support a framework in which methylglyoxal functions as a central cellular stress mediator, integrating protein glycation, proteostasis disruption, oxidative stress, metabolic dysfunction, and immune activation into a unified network of cellular damage. This integrated model provides a foundational understanding of how methylglyoxal may drive neuronal dysfunction in diabetes and DPN (Figure 2).

## 4 Methylglyoxal and the DRG: the Missing Link

Despite extensive research on methylglyoxal across various disease contexts and cell types, little work has examined its impact on DRG beyond nociception and pain. Methylglyoxal is estimated to be at a plasma concentration around 60–250 nM with intracellular levels estimated to be between 1 and 5 μM in healthy patients (Berdowska et al. 2023). However, it is elevated in the plasma of patients with diabetes, exceeding 800 nM (Bierhaus et al. 2012; Eberhardt et al. 2012; Huang et al. 2016). It is also a noxious agent and has become an important molecule in diabetes and DPN research due to its role in nociception. Methylglyoxal‐related nociception occurs through its activation of TRPA1 and modification of NAV1.8 (Eberhardt et al. 2012; Huang et al. 2016). Methylglyoxal contributes to the development and prolongation of chronic pain in DPN through neuronal sensitization (Andersson et al. 2013; Basu et al. 2025). Methylglyoxal can also induce axon degeneration, making it an effective tool for investigating the relationship between pain and nerve degeneration (Griggs et al. 2021; Yousuf et al. 2025). Additionally, it is an efficient model for investigating the mechanistic changes underlying DPN development.

In peripheral neuropathy, sensory neurons are affecected more than motor neurons, making the DRG a vital tissue to study peripheral neuropathy (Mauermann and Staff NP 2026). Peripheral afferent sensory neurons are highly vulnerable to degeneration in DPN, as DPN degeneration typically starts as small‐fiber neuropathy, in which smaller‐diameter neurons are initially affected. Additionally, DPN tends to be length‐dependent, with the longest axons affected first (Mauermann and Staff NP 2026). As the condition progresses, larger‐diameter neurons and shorter axons can also degenerate. As a result, nociceptors are often the first neuronal subtype affected in neuropathy (Kobayashi and Zochodne 2018).

DRG expresses the glucose transporters GLUT1 and GLUT3, which are not insulin‐mediated and are upregulated during hyperglycemia (Rawat and Morrison 2021; Ebeling et al. 1998). As a result, they are particularly vulnerable to high blood glucose levels in insulin‐resistant conditions, since insulin‐mediated cells are unable to take up glucose. Instead, high glucose levels readily enter peripheral nerves via non‐insulin‐mediated mechanisms. This increases glycolysis and leads to high levels of methylglyoxal accumulating in the DRG during diabetes. Additionally, GLO1 activity is impaired in diabetes (Rabbani and Thornalley 2014b; Rabbani and Thornalley 2019; Rasul et al. 2018; Pacal et al. 2018) and is reduced in peripheral nerves (Bierhaus et al. 2012), leaving DRG vulnerable to elevated methylglyoxal levels under hyperglycemic conditions.

Given the roles of both DRG and methylglyoxal in diabetes and DPN, determining whether the methylglyoxal effect observed in other cell types is also present in DRG could provide a better understanding of methylglyoxal's contribution to diabetes complications and DPN. Below, we discuss what is known about the five key mechanisms in DRG and whether methylglyoxal affects DRG neurons in similar ways.

### 4.1 Protein Glycation in DRG

Methylglyoxal is a potent glycating agent that has an affinity for lysine, arginine, and cysteine residues to form AGEs (Maessen et al. 2015; Donnellan et al. 2025; Rosca et al. 2005). Formation of AGEs results in altered protein structure, disrupted enzyme activity, impaired protein–protein interactions, and DNA damage (Donnellan et al. 2025; Yamagishi et al. 2015; Najjar and Calvert 2024). The accumulation of glycated proteins has been implicated in multiple neurodegenerative and metabolic disorders and is increasingly recognized as a contributor to peripheral neuropathy and axonal degeneration (Ott et al. 2014; Najjar and Calvert 2024; Vicente Miranda et al. 2016; Rabbani and Thornalley 2021; Thornalley 2002; Ryle et al. 1997).

There are various avenues through which protein glycation can lead to cellular stress within the DRG. AGEs have been observed in DRG and are attributed to playing a role in nociception (Liu et al. 2017; Bufalo et al. 2019). Cytoskeletal proteins, including tubulin and neurofilaments, have been observed to have increased protein glycation in diabetic rats within 8 months, which may interfere with axonal transport, neurite stability, and mitochondrial trafficking (Ryle et al. 1997). Glycation of mitochondrial proteins can impair mitochondria by disrupting the electron transport chain and ATP production, resulting in altered bioenergetics (Rosca et al. 2005; Naudi et al. 2010). Ultimately, this could be detrimental to the energetic demands required to maintain peripheral axons. Additionally, glycation of mitochondrial proteins may lead to increased oxidative stress, creating a positive feedback loop of cellular stress (Rosca et al. 2005). Protein glycation prevents protein degradation, leading to the accumulation of misfolded and aggregated proteins (Ott et al. 2014; Vicente Miranda et al. 2016). This could lead to disruption of proteostasis pathways, resulting in ER stress and ultimately, axon degeneration. AGEs are also known to drive neuroinflammation through activating RAGE in neurons. RAGE is expressed in both DRG and Schwann cells (Bufalo et al. 2019). RAGE activation leads to NF‐κB signaling, cytokine release, oxidative stress, and neuroinflammation‐associated axonal injury (Piras et al. 2016; Tobon‐Velasco et al. 2014). Protein glycation connects metabolic changes, oxidative stress, inflammation, and proteostasis disruption and warrants further investigation into methylglyoxal‐driven glycation in DRG as an initiator of the interconnected cellular stress pathways that contribute to neuronal dysfunction.

### 4.2 Oxidative Stress in DRG

Multiple studies have examined how methylglyoxal generates ROS via AGEs. ROS can induce nociception through TRP receptors (Ueno et al. 2024). Most of our understanding of methylglyoxal‐induced ROS in DRGs is within the context of TRP channel‐mediated nociception, in which TRPV1 activation increases ROS (Ma et al. 2009). Within DPN, research indicates that hyperglycemia increases oxidative stress in the DRG more than in the peripheral axons and Schwann cells (Schmeichel et al. 2003). There is a need to understand the mechanisms underlying methylglyoxal‐induced accumulation of ROS in DRG and its implications.

### 4.3 Immune Signaling in DRG

It is likely that the methylglyoxal‐induced pro‐inflammatory immune response observed in various cell types also occurs in DRG. DRGs also contain non‐neuronal support cells capable of secreting cytokines, including satellite glial cells, Schwann cells, and immune cells such as macrophages and lymphocytes. It is plausible that methylglyoxal induces a direct immune response within DRG neurons, but it is also very likely that it elicits an immune response in the non‐neuronal cells surrounding DRG that indirectly impact DRG neurons. This key cellular communication within the DRG remains to be explored in response to methylglyoxal. Additionally, many sensory neurons express TRPA1, an ion channel in nociceptors that is directly activated by methylglyoxal, as discussed above. TRPA1 is also a pronociceptive channel that responds to inflammatory mediators (Andersson et al. 2013), providing a clear pathway by which DRGs can respond to immune threats. Additionally, low concentrations of methylglyoxal in DRG activate the integrated stress response, a key mediator of immune cell function and inflammation (Barragan‐Iglesias et al. 2019). Taken together, the immune‐responsive microenvironment of the DRG, the presence of methylglyoxal‐sensitive pathways such as TRPA1, and evidence that methylglyoxal activates stress‐response signaling in these neurons suggest that methylglyoxal may contribute to inflammatory signaling within the DRG. It will be important to identify the cellular sources and communications underlying these effects to better design pharmacological treatments.

### 4.4 Proteostasis Changes in DRG

Proteostasis changes are a distinctive feature of neurodegenerative diseases, demyelinating peripheral neuropathy, and spinal cord injury, as they lead to the accumulation of dysfunctional proteins that can become cytotoxic (Libberecht et al. 2023). Interestingly, activation of the unfolded protein response promoted axonal regeneration in peripheral nerves following injury, indicating that proteostasis regulation is already an important factor in DRG in the context of peripheral neuropathy (Onate et al. 2016). Additionally, proteomic analyses in paclitaxel‐induced neuropathy and spinal nerve ligation models both showed significant protein changes in the DRG (Komori et al. 2007). Notably, in the paclitaxel model, 165 proteins were significantly altered following the same paradigm previously used by this research group to induce both thermal and mechanical hypersensitivity, further highlighting the link between proteostasis changes and painful neuropathy (Hanna, Graur, Sinclair, McKiver, et al. 2024). The proteomics analysis of spinal ligation demonstrated protein changes involved in host defense, antioxidant defense, metabolism, and cellular stress responses. Additionally, there was increased expression of proteins involved in actin cytoskeletal organization, suggesting cytoskeletal stabilization as a critical response to peripheral nerve injury. Protein changes in injured DRG were similar to prior ligation nerve injury studies, with the added data on changes in metabolic enzyme regulation. Ultimately, this study suggests the importance of metabolic regulation and proteostasis in the cellular response to peripheral nerve injury (Komori et al. 2007).

This data highlights the prevalence of proteostasis changes occurring in DRG in the context of neuropathy. Future research is needed to investigate whether methylglyoxal, within DPN, triggers a proteostasis stress response that contributes to neuropathy.

### 4.5 Metabolic Regulation in DRG

Research addressing acute and chronic pain has increasingly focused on the role of metabolism in the development and chronification of pain. Multiple groups have begun to link changes in cellular metabolism to pain (Willemen et al. 2023; Li et al. 2025). Additionally, metabolic changes appear to play a key role in axon degeneration (Sasaki 2019; Griffin and Ackerman 2020; Brull et al. 2025). Sarm1 is an enzyme involved in Wallerian degeneration and appears to act as a metabolic sensor, with its activity regulated by metabolic intermediates, including nicotinamide mononucleotide (NMN) and NAD+ (Figley et al. 2021). Given the growing interest in metabolic reprogramming in pain and axon degeneration, and the established role of methylglyoxal in altering cellular metabolism in other systems, it is critical to determine whether similar effects occur in DRG neurons. Defining methylglyoxal‐induced metabolic changes in DRG may provide novel insight into mechanisms of pain and SARM1‐mediated axon degeneration in diabetic peripheral neuropathy.

## 5 Therapeutic Strategies Targeting Methylglyoxal

Understanding the role of methylglyoxal in cellular metabolism in DRG creates new opportunities for therapeutic development. If methylglyoxal similarly induces oxidative stress and ROS accumulation in DRG, targeting oxidative stress could be a potential therapeutic approach. Multiple studies in vitro and in vivo have mitigated the effects of methylglyoxal through either reducing methylglyoxal‐induced oxidative stress or using an antioxidant (Wang et al. 2022; Wang, Chen, et al. 2024; Jarisarapurin et al. 2021; Nuamnaichati et al. 2020). However, multiple clinical trials targeting diabetic peripheral neuropathy with antioxidant interventions have not been effective (Sima et al. 1993; Tolrestat 2005; College A‐RU 2023; Santiago et al. 1993; Ziegler et al. 2011) (Table 1). It is logical to hypothesize that an increase in methylglyoxal, which decreases glutathione through the glyoxalase system, would increase ROS. Additionally, increasing glutathione could be a potential therapeutic route to explore. Using improved antioxidants to target the increased ROS induced by methylglyoxal could be a potential therapeutic approach. Previous work has suggested an intervention targeting the glyoxalase system (Kim et al. 2022; Rabbani and Thornalley 2022). A plausible therapeutic option is targeting AGEs, as they strongly contribute to ROS accumulation (Soulis et al. 1996). Potential interventions include aminoguanidine, which can effectively reduce the accumulation of AGEs but is hampered by toxic side effects (Thornalley 2003; Freedman et al. 1999). Identifying novel drugs with fewer side effects that target key cells will be essential to developing better clinical treatments.

Additionally, it is important to consider the limitations of the studies analyzed in this review. Although immortalized and transformed cell lines have provided important insight into the mechanism of methylglyoxal biology, transformed cell lines often exhibit altered metabolic states, stress responses, and gene expression compared with primary neurons and in vivo systems. As a result, there may be significant different responses to methylglyoxal‐induced glycation and downstream signaling pathways. Consequently, responses observed in such models may not fully reflect those occurring in primary sensory neurons or in vivo. Validation in primary neuronal cultures and animal models is therefore essential to support translational interpretation in the context of diabetic peripheral neuropathy. This further highlights the need to investigate the role of methylglyoxal in DRG and in vivo to understand its biological effects and identify potential mechanisms that could be targeted for novel DPN therapeutics.

## 6 Conclusion

DPN remains a highly prevalent and debilitating complication of diabetes with limited effective disease‐modifying therapies. While hyperglycemia has long been implicated, growing evidence highlights methylglyoxal as a key metabolic mediator, linking altered glucose metabolism to neuronal dysfunction. As a reactive by‐product of glycolysis, methylglyoxal accumulates in diabetes due to increased production and impaired detoxification, particularly in metabolically vulnerable DRG neurons. Beyond its emerging role in nociception, methylglyoxal exerts widespread effects through interconnected mechanisms: protein glycation, disruption of proteostasis, oxidative stress, mitochondrial and metabolic dysfunction, and activation of inflammatory signaling. These pathways form a self‐reinforcing network of cellular stress that likely contributes to both axonal degeneration and persistent pain in DPN. Despite extensive evidence in other tissues, the impact of these mechanisms in DRG neurons remains underexplored. Given the unique metabolic profile and anatomical vulnerability of DRG neurons, methylglyoxal accumulation may represent a critical and underappreciated cause of neuropathic progression and pain. Integrating these mechanistic insights with new and emerging information about pain‐ and axon‐degeneration‐associated signaling pathways will be critical for understanding how to pharmacologically address methylglyoxal's role in peripheral nerve dysfunction. We believe that targeting methylglyoxal production, detoxification pathways, or its downstream effects remains a promising therapeutic opportunity.

## Author Contributions

**Douglas E. Wright:** funding acquisition, writing – review and editing, resources, supervision. **Gentry Totta‐Griese:** conceptualization, investigation, writing – original draft, writing – review and editing, visualization.

## Funding

This work was supported by National Institutes of Health, R01NS043314, RO1NS144393. Kansas INBRE, P20GM1033418.

## Conflicts of Interest

The authors declare no conflicts of interest.

## Data Availability

The authors have nothing to report.

## References

[jnc70529-bib-0001] Allaman, I. , M. Belanger , and P. J. Magistretti . 2015. “Methylglyoxal, the Dark Side of Glycolysis.” Frontiers in Neuroscience 9: 23. 25709564 10.3389/fnins.2015.00023 PMC4321437

[jnc70529-bib-0002] Andersson, D. A. , C. Gentry , E. Light , et al. 2013. “Methylglyoxal Evokes Pain by Stimulating TRPA1.” PLoS One 8, no. 10: e77986. 24167592 10.1371/journal.pone.0077986 PMC3805573

[jnc70529-bib-0003] Apfel, S. C. , S. Schwartz , B. T. Adornato , et al. 2000. “Efficacy and Safety of Recombinant Human Nerve Growth Factor in Patients With Diabetic Polyneuropathy: A Randomized Controlled Trial. rhNGF Clinical Investigator Group.” Journal of the American Medical Association 284, no. 17: 2215–2221. 11056593 10.1001/jama.284.17.2215

[jnc70529-bib-0004] Azizian‐Farsani, F. , N. Abedpoor , M. Hasan Sheikhha , A. O. Gure , M. H. Nasr‐Esfahani , and K. Ghaedi . 2020. “Receptor for Advanced Glycation End Products Acts as a Fuel to Colorectal Cancer Development.” Frontiers in Oncology 10: 552283. 33117687 10.3389/fonc.2020.552283 PMC7551201

[jnc70529-bib-0005] Baltan, S. , D. M. Inman , C. A. Danilov , R. S. Morrison , D. J. Calkins , and P. J. Horner . 2010. “Metabolic Vulnerability Disposes Retinal Ganglion Cell Axons to Dysfunction in a Model of Glaucomatous Degeneration.” Journal of Neuroscience 30, no. 16: 5644–5652. 20410117 10.1523/JNEUROSCI.5956-09.2010 PMC2884009

[jnc70529-bib-0006] Barragan‐Iglesias, P. , J. Kuhn , G. C. Vidal‐Cantu , et al. 2019. “Activation of the Integrated Stress Response in Nociceptors Drives Methylglyoxal‐Induced Pain.” Pain 160, no. 1: 160–171. 30157134 10.1097/j.pain.0000000000001387 PMC6800106

[jnc70529-bib-0007] Bartling, B. , H. S. Hofmann , A. Sohst , et al. 2011. “Prognostic Potential and Tumor Growth‐Inhibiting Effect of Plasma Advanced Glycation End Products in Non‐Small Cell Lung Carcinoma.” Molecular Medicine 17, no. 9–10: 980–989. 21629968 10.2119/molmed.2011.00085 PMC3188878

[jnc70529-bib-0008] Basu, P. , D. F. S. Santos , N. Gakii , et al. 2025. “Long‐Lasting and Fast Methylglyoxal‐Scavenging Peptide CycK(Myr)R(4)E Alleviates Chronic Pain in Type 2 Diabetic Mice.” Pain Reports 10, no. 5: e1312. 40809797 10.1097/PR9.0000000000001312 PMC12348387

[jnc70529-bib-0009] Beirowski, B. 2022. “Emerging Evidence for Compromised Axonal Bioenergetics and Axoglial Metabolic Coupling as Drivers of Neurodegeneration.” Neurobiology of Disease 170: 105751. 35569720 10.1016/j.nbd.2022.105751

[jnc70529-bib-0010] Beisswenger, P. J. 2014. “Methylglyoxal in Diabetes: Link to Treatment, Glycaemic Control and Biomarkers of Complications.” Biochemical Society Transactions 42, no. 2: 450–456. 24646259 10.1042/BST20130275

[jnc70529-bib-0011] Bento, C. F. , F. Marques , R. Fernandes , and P. Pereira . 2010. “Methylglyoxal Alters the Function and Stability of Critical Components of the Protein Quality Control.” PLoS One 5, no. 9: e13007. 20885985 10.1371/journal.pone.0013007 PMC2945773

[jnc70529-bib-0012] Berdowska, I. , M. Matusiewicz , and I. Fecka . 2023. “Methylglyoxal in Cardiometabolic Disorders: Routes Leading to Pathology Counterbalanced by Treatment Strategies.” Molecules 28, no. 23: 7742. 38067472 10.3390/molecules28237742 PMC10708463

[jnc70529-bib-0013] Bhandari, R. , A. Sharma , and A. Kuhad . 2021. “Novel Nanotechnological Approaches for Targeting Dorsal Root Ganglion (DRG) in Mitigating Diabetic Neuropathic Pain (DNP).” Front Endocrinol (Lausanne) 12: 790747. 35211091 10.3389/fendo.2021.790747 PMC8862660

[jnc70529-bib-0014] Bhatti, J. S. , G. K. Bhatti , and P. H. Reddy . 2017. “Mitochondrial Dysfunction and Oxidative Stress in Metabolic Disorders–A Step Towards Mitochondria Based Therapeutic Strategies.” Biochimica et Biophysica Acta ‐ Molecular Basis of Disease 1863, no. 5: 1066–1077. 27836629 10.1016/j.bbadis.2016.11.010 PMC5423868

[jnc70529-bib-0015] Bierhaus, A. , T. Fleming , S. Stoyanov , et al. 2012. “Methylglyoxal Modification of Nav1.8 Facilitates Nociceptive Neuron Firing and Causes Hyperalgesia in Diabetic Neuropathy.” Nature Medicine 18, no. 6: 926–933. 10.1038/nm.2750 22581285

[jnc70529-bib-0016] Bonhof, G. J. , G. Sipola , A. Strom , et al. 2022. “BOND Study: A Randomised Double‐Blind, Placebo‐Controlled Trial Over 12 Months to Assess the Effects of Benfotiamine on Morphometric, Neurophysiological and Clinical Measures in Patients With Type 2 Diabetes With Symptomatic Polyneuropathy.” BMJ Open 12, no. 2: e057142. 10.1136/bmjopen-2021-057142 PMC8814806 35115359

[jnc70529-bib-0017] Brull, M. , S. Multrus , M. Schafer , I. Celardo , C. Karreman , and M. Leist . 2025. “Programmed Neurite Degeneration in Human Central Nervous System Neurons Driven by Changes in NAD^+^ Metabolism.” Cell Death & Disease 16, no. 1: 24. 39824831 10.1038/s41419-024-07326-w PMC11742042

[jnc70529-bib-0018] Bufalo, M. C. , M. E. Almeida , I. A. Franca , et al. 2019. “Advanced Glycation Endproducts Produced by In Vitro Glycation of Type I Collagen Modulate the Functional and Secretory Behavior of Dorsal Root Ganglion Cells Cultivated in Two‐Dimensional System.” Experimental Cell Research 382, no. 2: 111475. 31255600 10.1016/j.yexcr.2019.06.020

[jnc70529-bib-0019] Chen, H. , Y. Li , Y. Zhu , et al. 2017. “Advanced Glycation End Products Promote ChREBP Expression and Cell Proliferation in Liver Cancer Cells by Increasing Reactive Oxygen Species.” Medicine (Baltimore) 96, no. 33: e7456. 28816938 10.1097/MD.0000000000007456 PMC5571675

[jnc70529-bib-0020] Chen, W. , W. Huang , Y. Yang , and K. Li . 2022. “Methylglyoxal Scavengers Attenuate Angiogenesis Dysfunction Induced by Methylglyoxal and Oxygen‐Glucose Deprivation.” Oxidative Medicine and Cellular Longevity 2022: 8854457. 35035668 10.1155/2022/8854457 PMC8754597

[jnc70529-bib-0021] Chen, Y. H. , Z. W. Chen , H. M. Li , X. F. Yan , and B. Feng . 2018. “AGE/RAGE‐Induced EMP Release via the NOX‐Derived ROS Pathway.” Journal of Diabetes Research 2018: 6823058. 29744367 10.1155/2018/6823058 PMC5878883

[jnc70529-bib-0022] Cheng, R. X. , Y. Feng , D. Liu , et al. 2019. “The Role of Na(v)1.7 and Methylglyoxal‐Mediated Activation of TRPA1 in Itch and Hypoalgesia in a Murine Model of Type 1 Diabetes.” Theranostics 9, no. 15: 4287–4307. 31285762 10.7150/thno.36077 PMC6599654

[jnc70529-bib-0023] Chiavarina, B. , M. J. Nokin , F. Durieux , et al. 2014. “Triple Negative Tumors Accumulate Significantly Less Methylglyoxal Specific Adducts Than Other Human Breast Cancer Subtypes.” Oncotarget 5, no. 14: 5472–5482. 24978626 10.18632/oncotarget.2121 PMC4170620

[jnc70529-bib-0024] Choi, J. , S. Tak , H. M. Jung , et al. 2023. “Kinetic Evidence in Favor of Glyoxalase III and Against Deglycase Activity of DJ‐1.” Protein Science 32, no. 5: e4641. 37060572 10.1002/pro.4641 PMC10127264

[jnc70529-bib-0025] Cianfruglia, L. , C. Morresi , T. Bacchetti , T. Armeni , and G. Ferretti . 2020. “Protection of Polyphenols Against Glyco‐Oxidative Stress: Involvement of Glyoxalase Pathway.” Antioxidants (Basel) 9, no. 10: 1006. 33081239 10.3390/antiox9101006 PMC7602851

[jnc70529-bib-0026] Coccini, T. , A. Schicchi , C. A. Locatelli , et al. 2023. “Methylglyoxal‐Induced Neurotoxic Effects in Primary Neuronal‐Like Cells Transdifferentiated From Human Mesenchymal Stem Cells: Impact of Low Concentrations.” Journal of Applied Toxicology 43, no. 12: 1819–1839. 37431083 10.1002/jat.4515

[jnc70529-bib-0027] College A‐RU . 2023. “Pyridoxine Effect on the Blood Glucose Level in Type 2 Diabetic Patients ClinicalTrials.gov: National Library of Medicine Clinical Trial ID: NCT05918068.” https://clinicaltrials.gov/study/NCT05918068?tab=researcher#trial‐contacts.

[jnc70529-bib-0028] Cortizo, F. G. , D. Pfaff , A. Wirth , et al. 2022. “The Activity of Glyoxylase 1 Is Regulated by Glucose‐Responsive Phosphorylation on Tyr136.” Molecular Metabolism 55: 101406. 34838714 10.1016/j.molmet.2021.101406 PMC8715127

[jnc70529-bib-0029] Darmaun, D. , S. D. Smith , S. Sweeten , B. K. Sager , S. Welch , and N. Mauras . 2005. “Evidence for Accelerated Rates of Glutathione Utilization and Glutathione Depletion in Adolescents With Poorly Controlled Type 1 Diabetes.” Diabetes 54, no. 1: 190–196. 15616028 10.2337/diabetes.54.1.190

[jnc70529-bib-0030] Dave, V. , and Y. Patel . 2025. “Detection of Diabetic Peripheral Neuropathy From Index Finger Using Vibration Mechanism.” Journal of Medical Engineering & Technology 49, no. 5: 171–178. 40418141 10.1080/03091902.2025.2508229

[jnc70529-bib-0031] de Bari, L. , A. Scire , C. Minnelli , L. Cianfruglia , M. P. Kalapos , and T. Armeni . 2020. “Interplay Among Oxidative Stress, Methylglyoxal Pathway and S‐Glutathionylation.” Antioxidants 10, no. 1: 19. 33379155 10.3390/antiox10010019 PMC7824032

[jnc70529-bib-0032] Deng, X. , Q. Wu , and Y. Liu . 2024. “Eucommia ulmoidesOliv. Leaves Flavonoids Attenuate Methylglyoxal‐Induced Endothelial Cell Apoptosis In Vitro and In Vivo by Upregulating AKT‐Nrf2 Signaling and Downregulating Oxidative Stress.” Food Science & Nutrition 12, no. 10: 7938–7953. 39479661 10.1002/fsn3.4416 PMC11521679

[jnc70529-bib-0033] Donnellan, L. , M. Fenech , V. S. Dhillon , C. Young , P. Hoffmann , and P. Deo . 2025. “Role of Methylglyoxal Protein Modifications in DNA Damage and Chromosomal Instability: Emerging Molecular Mechanisms.” Mutation Research, Reviews in Mutation Research 796: 108558. 40779948 10.1016/j.mrrev.2025.108558

[jnc70529-bib-0034] Doty, M. , S. Yun , Y. Wang , et al. 2022. “Integrative Multiomic Analyses of Dorsal Root Ganglia in Diabetic Neuropathic Pain Using Proteomics, Phospho‐Proteomics, and Metabolomics.” Scientific Reports 12, no. 1: 17012. 36220867 10.1038/s41598-022-21394-y PMC9553906

[jnc70529-bib-0035] Ebeling, P. , H. A. Koistinen , and V. A. Koivisto . 1998. “Insulin‐Independent Glucose Transport Regulates Insulin Sensitivity.” FEBS Letters 436, no. 3: 301–303. 9801136 10.1016/s0014-5793(98)01149-1

[jnc70529-bib-0036] Eberhardt, M. J. , M. R. Filipovic , A. Leffler , et al. 2012. “Methylglyoxal Activates Nociceptors Through Transient Receptor Potential Channel A1 (TRPA1): A Possible Mechanism of Metabolic Neuropathies.” Journal of Biological Chemistry 287, no. 34: 28291–28306. 22740698 10.1074/jbc.M111.328674 PMC3436587

[jnc70529-bib-0037] Egawa, T. , T. Ogawa , T. Yokokawa , K. Kido , K. Goto , and T. Hayashi . 2022. “Methylglyoxal Reduces Molecular Responsiveness to 4 Weeks of Endurance Exercise in Mouse Plantaris Muscle.” Journal of Applied Physiology (1985) 132, no. 2: 477–488. 10.1152/japplphysiol.00539.2021 35023763

[jnc70529-bib-0038] Enders, J. D. , S. Thomas , M. T. Swanson , J. M. Ryals , and D. E. Wright . 2022. “Ketogenic Diet Prevents Methylglyoxal‐Evoked Nociception by Scavenging Methylglyoxal.” Pain 163, no. 12: e1207–e1216. 35500286 10.1097/j.pain.0000000000002667 PMC9727824

[jnc70529-bib-0039] Figley, M. D. , W. Gu , J. D. Nanson , et al. 2021. “SARM1 Is a Metabolic Sensor Activated by an Increased NMN/NAD^+^ Ratio to Trigger Axon Degeneration.” Neuron 109, no. 7: 1118‐36. 33657413 10.1016/j.neuron.2021.02.009 PMC8174188

[jnc70529-bib-0040] Fournet, M. , F. Bonte , and A. Desmouliere . 2018. “Glycation Damage: A Possible Hub for Major Pathophysiological Disorders and Aging.” Aging and Disease 9, no. 5: 880–900. 30271665 10.14336/AD.2017.1121 PMC6147582

[jnc70529-bib-0041] Fraser, D. A. , L. M. Diep , I. A. Hovden , et al. 2012. “The Effects of Long‐Term Oral Benfotiamine Supplementation on Peripheral Nerve Function and Inflammatory Markers in Patients With Type 1 Diabetes: A 24‐Month, Double‐Blind, Randomized, Placebo‐Controlled Trial.” Diabetes Care 35, no. 5: 1095–1097. 22446172 10.2337/dc11-1895 PMC3329837

[jnc70529-bib-0042] Freedman, B. I. , J. P. Wuerth , K. Cartwright , et al. 1999. “Design and Baseline Characteristics for the Aminoguanidine Clinical Trial in Overt Type 2 Diabetic Nephropathy (ACTION II).” Controlled Clinical Trials 20, no. 5: 493–510. 10503809 10.1016/s0197-2456(99)00024-0

[jnc70529-bib-0043] Giovannucci, E. , D. M. Harlan , M. C. Archer , et al. 2010. “Diabetes and Cancer: A Consensus Report.” Diabetes Care 33, no. 7: 1674–1685. 20587728 10.2337/dc10-0666 PMC2890380

[jnc70529-bib-0044] Griffin, E. N. , and S. L. Ackerman . 2020. “Lipid Metabolism and Axon Degeneration: An ACOX1 Balancing Act.” Neuron 106, no. 4: 551–553. 32437651 10.1016/j.neuron.2020.04.030

[jnc70529-bib-0045] Griffith, H. R. , J. A. den Hollander , O. C. Okonkwo , T. O'Brien , R. L. Watts , and D. C. Marson . 2008. “Brain Metabolism Differs in Alzheimer's Disease and Parkinson's Disease Dementia.” Alzheimer's & Dementia 4, no. 6: 421–427. 10.1016/j.jalz.2008.04.008 PMC2600665 19012867

[jnc70529-bib-0046] Griggs, R. B. , D. E. Laird , R. R. Donahue , W. Fu , and B. K. Taylor . 2017. “Methylglyoxal Requires AC1 and TRPA1 to Produce Pain and Spinal Neuron Activation.” Frontiers in Neuroscience 11: 679. 29270106 10.3389/fnins.2017.00679 PMC5723675

[jnc70529-bib-0047] Griggs, R. B. , D. V. M. Nguyen , L. M. Yermakov , et al. 2021. “The Type 2 Diabetes Factor Methylglyoxal Mediates Axon Initial Segment Shortening and Alters Neuronal Function at the Cellular and Network Levels.” eNeuro 8, no. 5: ENEU21.2021. 10.1523/ENEURO.0201-21.2021 PMC8496204 34531281

[jnc70529-bib-0048] Griggs, R. B. , L. M. Yermakov , D. E. Drouet , D. V. M. Nguyen , and K. Susuki . 2018. “Methylglyoxal Disrupts Paranodal Axoglial Junctions via Calpain Activation.” ASN Neuro 10: 1759091418766175. 29673258 10.1177/1759091418766175 PMC5944142

[jnc70529-bib-0049] Hanna, R. , A. Graur , P. Sinclair , et al. 2024. “Proteomic Analysis of Dorsal Root Ganglia in a Mouse Model of Paclitaxel‐Induced Neuropathic Pain.” PLoS One 19, no. 9: e0306498. 39331687 10.1371/journal.pone.0306498 PMC11432834

[jnc70529-bib-0051] Hanssen, N. M. , C. D. Stehouwer , and C. G. Schalkwijk . 2014. “Methylglyoxal and Glyoxalase I in Atherosclerosis.” Biochemical Society Transactions 42, no. 2: 443–449. 24646258 10.1042/BST20140001

[jnc70529-bib-0052] Hanssen, N. M. J. , C. Tikellis , R. J. Pickering , et al. 2023. “Pyridoxamine Prevents Increased Atherosclerosis by Intermittent Methylglyoxal Spikes in the Aortic Arches of ApoE(−/−) Mice.” Biomedicine & Pharmacotherapy 158: 114211. 36916437 10.1016/j.biopha.2022.114211

[jnc70529-bib-0053] Harun‐Or‐Rashid, M. , N. Pappenhagen , P. G. Palmer , et al. 2018. “Structural and Functional Rescue of Chronic Metabolically Stressed Optic Nerves Through Respiration.” Journal of Neuroscience 38, no. 22: 5122–5139. 29760184 10.1523/JNEUROSCI.3652-17.2018 PMC5977447

[jnc70529-bib-0054] Huang, Q. , Y. Chen , N. Gong , and Y. X. Wang . 2016. “Methylglyoxal Mediates Streptozotocin‐Induced Diabetic Neuropathic Pain via Activation of the Peripheral TRPA1 and Nav1.8 Channels.” Metabolism 65, no. 4: 463–474. 26975538 10.1016/j.metabol.2015.12.002

[jnc70529-bib-0055] Huang, X. , F. Wang , W. Chen , Y. Chen , N. Wang , and K. von Maltzan . 2012. “Possible Link Between the Cognitive Dysfunction Associated With Diabetes Mellitus and the Neurotoxicity of Methylglyoxal.” Brain Research 1469: 82–91. 22750288 10.1016/j.brainres.2012.06.011

[jnc70529-bib-0056] Hurben, A. K. , Q. Zhang , J. J. Galligan , N. Tretyakova , and L. Erber . 2024. “Endogenous Cellular Metabolite Methylglyoxal Induces DNA‐Protein Cross‐Links in Living Cells.” ACS Chemical Biology 19, no. 6: 1291–1302. 38752800 10.1021/acschembio.4c00100 PMC11353540

[jnc70529-bib-0057] IARC . 1991. “IARC Working Group on the Evaluation of Carcinogenic Risks to Humans.” In Coffee, Tea, Mate, Methylxanthines and Methylglyoxal, vol. 51, 1–513. IARC. PMC7681554 1674554

[jnc70529-bib-0058] International Diabetes Federation . 2026. “Facts and Figures: International Diabetes Federation.” https://idf.org/about‐diabetes/diabetes‐facts‐figures/.

[jnc70529-bib-0059] Jarisarapurin, W. , K. Kunchana , L. Chularojmontri , and S. K. Wattanapitayakul . 2021. “Unripe *Carica papaya* Protects Methylglyoxal‐Invoked Endothelial Cell Inflammation and Apoptosis via the Suppression of Oxidative Stress and Akt/MAPK/NF‐kappaB Signals.” Antioxidants (Basel) 10, no. 8: 1158. 34439407 10.3390/antiox10081158 PMC8388906

[jnc70529-bib-0060] Jun, Y. W. , and E. T. Kool . 2020. “Small Substrate or Large? Debate Over the Mechanism of Glycation Adduct Repair by DJ‐1.” Cell Chemical Biology 27, no. 9: 1117–1123. 32783963 10.1016/j.chembiol.2020.07.016 PMC8442549

[jnc70529-bib-0061] Kang, R. , D. Tang , M. T. Lotze , and H. J. Zeh 3rd . 2012. “AGER/RAGE‐Mediated Autophagy Promotes Pancreatic Tumorigenesis and Bioenergetics Through the IL6‐pSTAT3 Pathway.” Autophagy 8, no. 6: 989–991. 22722139 10.4161/auto.20258 PMC3427269

[jnc70529-bib-0062] Kessler, J. A. , A. Shaibani , C. N. Sang , et al. 2021. “Gene Therapy for Diabetic Peripheral Neuropathy: A Randomized, Placebo‐Controlled Phase III Study of VM202, a Plasmid DNA Encoding Human Hepatocyte Growth Factor.” Clinical and Translational Science 14, no. 3: 1176–1184. 33465273 10.1111/cts.12977 PMC8212761

[jnc70529-bib-0063] Kim, D. , J. Cheon , H. Yoon , and H. S. Jun . 2021. “ *Cudrania tricuspidata* Root Extract Prevents Methylglyoxal‐Induced Inflammation and Oxidative Stress via Regulation of the PKC‐NOX4 Pathway in Human Kidney Cells.” Oxidative Medicine and Cellular Longevity 2021: 5511881. 33859775 10.1155/2021/5511881 PMC8026309

[jnc70529-bib-0064] Kim, D. , K. A. Kim , J. H. Kim , E. H. Kim , and O. N. Bae . 2020. “Methylglyoxal‐Induced Dysfunction in Brain Endothelial Cells via the Suppression of Akt/HIF‐1alpha Pathway and Activation of Mitophagy Associated With Increased Reactive Oxygen Species.” Antioxidants (Basel) 9, no. 9: 820. 32899154 10.3390/antiox9090820 PMC7554889

[jnc70529-bib-0065] Kim, J. Y. , J. H. Jung , S. J. Lee , S. S. Han , and S. H. Hong . 2022. “Glyoxalase 1 as a Therapeutic Target in Cancer and Cancer Stem Cells.” Molecules and Cells 45, no. 12: 869–876. 36172978 10.14348/molcells.2022.0109 PMC9794553

[jnc70529-bib-0066] Kobayashi, M. , and D. W. Zochodne . 2018. “Diabetic Neuropathy and the Sensory Neuron: New Aspects of Pathogenesis and Their Treatment Implications.” Journal of Diabetes Investigation 9, no. 6: 1239–1254. 29533535 10.1111/jdi.12833 PMC6215951

[jnc70529-bib-0067] Komori, N. , N. Takemori , H. K. Kim , et al. 2007. “Proteomics Study of Neuropathic and Nonneuropathic Dorsal Root Ganglia: Altered Protein Regulation Following Segmental Spinal Nerve Ligation Injury.” Physiological Genomics 29, no. 2: 215–230. 17213366 10.1152/physiolgenomics.00255.2006

[jnc70529-bib-0068] Kong, L. R. , K. Gupta , A. J. Wu , et al. 2024. “A Glycolytic Metabolite Bypasses “Two‐Hit” Tumor Suppression by BRCA2.” Cell 187, no. 9: 2269–2287. 38608703 10.1016/j.cell.2024.03.006

[jnc70529-bib-0069] Lai, S. W. T. , C. Hernandez‐Castillo , E. J. L. Gonzalez , et al. 2024. “Methylglyoxal Adducts Are Prognostic Biomarkers for Diabetic Kidney Disease in Patients With Type 1 Diabetes.” Diabetes 73, no. 4: 611–617. 37967313 10.2337/db23-0277 PMC10958582

[jnc70529-bib-0070] Lai, S. W. T. , E. J. Lopez Gonzalez , T. Zoukari , P. Ki , and S. C. Shuck . 2022. “Methylglyoxal and Its Adducts: Induction, Repair, and Association With Disease.” Chemical Research in Toxicology 35, no. 10: 1720–1746. 36197742 10.1021/acs.chemrestox.2c00160 PMC9580021

[jnc70529-bib-0071] Li, X. , W. Bakker , Y. Sang , and I. Rietjens . 2024. “Absorption and Intracellular Accumulation of Food‐Borne Dicarbonyl Precursors of Advanced Glycation End‐Product in a Caco‐2 Human Cell Transwell Model.” Food Chemistry 452: 139532. 38705120 10.1016/j.foodchem.2024.139532

[jnc70529-bib-0072] Li, X. , Z. Zhao , Y. Ke , Y. Jiang , Y. Liu , and Z. Liu . 2025. “Links Between Cellular Energy Metabolism and Pain Sensation.” Anesthesia and Analgesia 140, no. 3: 616–627. 39110636 10.1213/ANE.0000000000007096 PMC11805490

[jnc70529-bib-0073] Libberecht, K. , T. Vangansewinkel , L. Van Den Bosch , I. Lambrichts , and E. Wolfs . 2023. “Proteostasis Plays an Important Role in Demyelinating Charcot Marie Tooth Disease.” Biochemical Pharmacology 216: 115760. 37604292 10.1016/j.bcp.2023.115760

[jnc70529-bib-0074] Liccardo, M. , L. Sapio , S. Perrella , I. Sirangelo , and C. Iannuzzi . 2024. “Genistein Prevents Apoptosis and Oxidative Stress Induced by Methylglyoxal in Endothelial Cells.” Molecules 29, no. 8: 1712. 38675531 10.3390/molecules29081712 PMC11052514

[jnc70529-bib-0075] Lin, J. A. , C. H. Wu , and G. C. Yen . 2018. “Methylglyoxal Displays Colorectal Cancer‐Promoting Properties in the Murine Models of Azoxymethane and CT26 Isografts.” Free Radical Biology & Medicine 115: 436–446. 29269310 10.1016/j.freeradbiomed.2017.12.020

[jnc70529-bib-0076] Liu, C. C. , X. S. Zhang , Y. T. Ruan , et al. 2017. “Accumulation of Methylglyoxal Increases the Advanced Glycation End‐Product Levels in DRG and Contributes to Lumbar Disk Herniation‐Induced Persistent Pain.” Journal of Neurophysiology 118, no. 2: 1321–1328. 28615337 10.1152/jn.00745.2016 PMC5558033

[jnc70529-bib-0077] Loreto, A. , K. M. L. Cramb , L. A. McDermott , et al. 2025. “SARM1 Activation Induces Reversible Mitochondrial Dysfunction and Can Be Prevented in Human Neurons by Antisense Oligonucleotides.” Neurobiology of Disease 213: 106986. 40473123 10.1016/j.nbd.2025.106986 PMC7617922

[jnc70529-bib-0078] Ma, F. , L. Zhang , and K. N. Westlund . 2009. “Reactive Oxygen Species Mediate TNFR1 Increase After TRPV1 Activation in Mouse DRG Neurons.” Molecular Pain 5: 31. 19531269 10.1186/1744-8069-5-31 PMC2706230

[jnc70529-bib-0079] Maessen, D. E. , C. D. Stehouwer , and C. G. Schalkwijk . 2015. “The Role of Methylglyoxal and the Glyoxalase System in Diabetes and Other Age‐Related Diseases.” Clinical Science (London, England: 1973) 128, no. 12: 839–861. 10.1042/CS20140683 25818485

[jnc70529-bib-0080] Masterjohn, C. , E. Mah , Y. Park , et al. 2013. “Acute Glutathione Depletion Induces Hepatic Methylglyoxal Accumulation by Impairing Its Detoxification to D‐Lactate.” Experimental Biology and Medicine (Maywood, N.J.) 238, no. 4: 360–369. 10.1177/1535370213477987 23760001

[jnc70529-bib-0081] Mauermann, M. L. , and Staff NP . 2026. “Peripheral Neuropathy: A Review.” Journal of the American Medical Association 335, no. 3: 255–266. 41247746 10.1001/jama.2025.19400

[jnc70529-bib-0082] Medeiros, M. L. , A. L. Oliveira , M. G. de Oliveira , F. Z. Monica , and E. Antunes . 2021. “Methylglyoxal Exacerbates Lipopolysaccharide‐Induced Acute Lung Injury via RAGE‐Induced ROS Generation: Protective Effects of Metformin.” Journal of Inflammation Research 14: 6477–6489. 34880648 10.2147/JIR.S337115 PMC8648108

[jnc70529-bib-0083] Mian, S. M. , S. I. Nakisli , B. J. Woodall , et al. 2025. “Eukaryotic Initiation Factor 3d Regulates Context‐Dependent Pain Hypersensitivity Through the Integrated Stress Response.” bioRxiv. 10.1016/j.ynpai.2026.100218 PMC13213694 42212231

[jnc70529-bib-0084] Miyashita, A. , M. Kobayashi , T. Yokota , and D. W. Zochodne . 2023. “Diabetic Polyneuropathy: New Strategies to Target Sensory Neurons in Dorsal Root Ganglia.” International Journal of Molecular Sciences 24, no. 6: 5977. 36983051 10.3390/ijms24065977 PMC10051459

[jnc70529-bib-0085] Murata, H. , M. Sakaguchi , K. Kataoka , and N. H. Huh . 2013. “SARM1 and TRAF6 Bind to and Stabilize PINK1 on Depolarized Mitochondria.” Molecular Biology of the Cell 24, no. 18: 2772–2784. 23885119 10.1091/mbc.E13-01-0016 PMC3771941

[jnc70529-bib-0086] Murata‐Kamiya, N. , and H. Kamiya . 2001. “Methylglyoxal, an Endogenous Aldehyde, Crosslinks DNA Polymerase and the Substrate DNA.” Nucleic Acids Research 29, no. 16: 3433–3438. 11504881 10.1093/nar/29.16.3433 PMC55850

[jnc70529-bib-0087] Najjar, J. A. , and J. W. Calvert . 2024. “Effects of Protein Glycation and Protective Mechanisms Against Glycative Stress.” Current Opinion in Pharmacology 76: 102464. 38796877 10.1016/j.coph.2024.102464 PMC11229435

[jnc70529-bib-0088] Nakamura, N. , T. Matsui , Y. Ishibashi , et al. 2017. “RAGE‐Aptamer Attenuates the Growth and Liver Metastasis of Malignant Melanoma in Nude Mice.” Molecular Medicine 23: 295–306. 29387865 10.2119/molmed.2017.00099 PMC5681699

[jnc70529-bib-0089] Naudi, A. , M. Jove , V. Ayala , M. Portero‐Otin , and R. Pamplona . 2010. “Glycation of Mitochondrial Proteins, Oxidative Stress and Aging.” Revista Española de Geriatría y Gerontología 45, no. 3: 156–166. 20347183 10.1016/j.regg.2010.02.001

[jnc70529-bib-0090] Nigro, C. , A. Leone , G. A. Raciti , et al. 2017. “Methylglyoxal‐Glyoxalase 1 Balance: The Root of Vascular Damage.” International Journal of Molecular Sciences 18, no. 1: 188. 28106778 10.3390/ijms18010188 PMC5297820

[jnc70529-bib-0091] Nuamnaichati, N. , S. Mangmool , N. Chattipakorn , and W. Parichatikanond . 2020. “Stimulation of GLP‐1 Receptor Inhibits Methylglyoxal‐Induced Mitochondrial Dysfunctions in H9c2 Cardiomyoblasts: Potential Role of Epac/PI3K/Akt Pathway.” Frontiers in Pharmacology 11: 805. 32547400 10.3389/fphar.2020.00805 PMC7274035

[jnc70529-bib-0092] Onate, M. , A. Catenaccio , G. Martinez , et al. 2016. “Activation of the Unfolded Protein Response Promotes Axonal Regeneration After Peripheral Nerve Injury.” Scientific Reports 6: 21709. 26906090 10.1038/srep21709 PMC4764858

[jnc70529-bib-0093] Osonoi, S. , H. Mizukami , Y. Takeuchi , et al. 2022. “RAGE Activation in Macrophages and Development of Experimental Diabetic Polyneuropathy.” JCI Insight 7, no. 23: e160555. 36477360 10.1172/jci.insight.160555 PMC9746912

[jnc70529-bib-0094] Ott, C. , K. Jacobs , E. Haucke , A. Navarrete Santos , T. Grune , and A. Simm . 2014. “Role of Advanced Glycation End Products in Cellular Signaling.” Redox Biology 2: 411–429. 24624331 10.1016/j.redox.2013.12.016 PMC3949097

[jnc70529-bib-0095] Pacal, L. , K. Chalasova , A. Pleskacova , J. Rehorova , J. Tomandl , and K. Kankova . 2018. “Deleterious Effect of Advanced CKD on Glyoxalase System Activity Not Limited to Diabetes Aetiology.” International Journal of Molecular Sciences 19, no. 5: 1517. 29783710 10.3390/ijms19051517 PMC5983829

[jnc70529-bib-0096] Parsons, K. , H. Yin , O. H. Y. Yu , F. Khosrow‐Khavar , and L. Azoulay . 2026. “The Association Between Pre‐Existing Type 2 Diabetes on Cancer‐Related and All‐Cause Mortality Among Women With Breast Cancer.” Breast Cancer Research and Treatment 216, no. 1: 3. 41661345 10.1007/s10549-026-07913-9

[jnc70529-bib-0097] Petersen, E. A. , T. G. Stauss , J. A. Scowcroft , et al. 2021. “Effect of High‐Frequency (10‐kHz) Spinal Cord Stimulation in Patients With Painful Diabetic Neuropathy: A Randomized Clinical Trial.” JAMA Neurology 78, no. 6: 687–698. 33818600 10.1001/jamaneurol.2021.0538 PMC8022268

[jnc70529-bib-0098] Piras, S. , A. L. Furfaro , C. Domenicotti , et al. 2016. “RAGE Expression and ROS Generation in Neurons: Differentiation Versus Damage.” Oxidative Medicine and Cellular Longevity 2016: 9348651. 27313835 10.1155/2016/9348651 PMC4897723

[jnc70529-bib-0099] Prantner, D. , S. Nallar , K. Richard , D. Spiegel , K. D. Collins , and S. N. Vogel . 2021. “Classically Activated Mouse Macrophages Produce Methylglyoxal That Induces a TLR4‐ and RAGE‐Independent Proinflammatory Response.” Journal of Leukocyte Biology 109, no. 3: 605–619. 32678947 10.1002/JLB.3A0520-745RR PMC7855181

[jnc70529-bib-0100] Prantner, D. , and S. N. Vogel . 2025. “Intracellular Methylglyoxal Accumulation in Classically Activated Mouse Macrophages Is Mediated by HIF‐1alpha.” Journal of Leukocyte Biology 117, no. 3. 10.1093/jleuko/qiae215 PMC12854256 39360990

[jnc70529-bib-0101] Price, C. L. , and S. C. Knight . 2007. “Advanced Glycation: A Novel Outlook on Atherosclerosis.” Current Pharmaceutical Design 13, no. 36: 3681–3687. 18220806 10.2174/138161207783018608

[jnc70529-bib-0102] Queisser, M. A. , D. Yao , S. Geisler , et al. 2010. “Hyperglycemia Impairs Proteasome Function by Methylglyoxal.” Diabetes 59, no. 3: 670–678. 20009088 10.2337/db08-1565 PMC2828656

[jnc70529-bib-0103] Rabbani, N. , and P. J. Thornalley . 2014a. “Dicarbonyl Proteome and Genome Damage in Metabolic and Vascular Disease.” Biochemical Society Transactions 42, no. 2: 425–432. 24646255 10.1042/BST20140018

[jnc70529-bib-0104] Rabbani, N. , and P. J. Thornalley . 2014b. “The Critical Role of Methylglyoxal and Glyoxalase 1 in Diabetic Nephropathy.” Diabetes 63, no. 1: 50–52. 24357696 10.2337/db13-1606

[jnc70529-bib-0105] Rabbani, N. , and P. J. Thornalley . 2019. “Glyoxalase 1 Modulation in Obesity and Diabetes.” Antioxidants & Redox Signaling 30, no. 3: 354–374. 29160087 10.1089/ars.2017.7424

[jnc70529-bib-0106] Rabbani, N. , and P. J. Thornalley . 2021. “Protein Glycation ‐ Biomarkers of Metabolic Dysfunction and Early‐Stage Decline in Health in the Era of Precision Medicine.” Redox Biology 42: 101920. 33707127 10.1016/j.redox.2021.101920 PMC8113047

[jnc70529-bib-0107] Rabbani, N. , and P. J. Thornalley . 2022. “Emerging Glycation‐Based Therapeutics‐Glyoxalase 1 Inducers and Glyoxalase 1 Inhibitors.” International Journal of Molecular Sciences 23, no. 5: 2453. 35269594 10.3390/ijms23052453 PMC8910005

[jnc70529-bib-0108] Rabbani, N. , M. Xue , M. O. Weickert , and P. J. Thornalley . 2018. “Multiple Roles of Glyoxalase 1‐Mediated Suppression of Methylglyoxal Glycation in Cancer Biology‐Involvement in Tumour Suppression, Tumour Growth, Multidrug Resistance and Target for Chemotherapy.” Seminars in Cancer Biology 49: 83–93. 28506645 10.1016/j.semcancer.2017.05.006

[jnc70529-bib-0109] Ramasamy, R. , S. F. Yan , and A. M. Schmidt . 2006. “Methylglyoxal Comes of AGE.” Cell 124, no. 2: 258–260. 16439200 10.1016/j.cell.2006.01.002

[jnc70529-bib-0110] Rasul, A. , A. Rashid , P. Waheed , and S. A. Khan . 2018. “Expression Analysis of Glyoxalase I Gene Among Patients of Diabetic Retinopathy.” Pakistan Journal of Medical Sciences 34, no. 1: 139–143. 29643895 10.12669/pjms.341.13839 PMC5856999

[jnc70529-bib-0111] Rawat, A. , and B. M. Morrison . 2021. “Metabolic Transporters in the Peripheral Nerve‐What, Where, and Why?” Neurotherapeutics 18, no. 4: 2185–2199. 34773210 10.1007/s13311-021-01150-2 PMC8804006

[jnc70529-bib-0112] Reddy, V. P. , P. Aryal , and P. Soni . 2023. “RAGE Inhibitors in Neurodegenerative Diseases.” Biomedicine 11, no. 4: 1131. 10.3390/biomedicines11041131 PMC10136230 37189749

[jnc70529-bib-0113] Rodrigues, D. C. , E. M. Harvey , R. Suraj , et al. 2020. “Methylglyoxal Couples Metabolic and Translational Control of Notch Signalling in Mammalian Neural Stem Cells.” Nature Communications 11, no. 1: 2018. 10.1038/s41467-020-15941-2 PMC7181744 32332750

[jnc70529-bib-0114] Rosca, M. G. , T. G. Mustata , M. T. Kinter , et al. 2005. “Glycation of Mitochondrial Proteins From Diabetic Rat Kidney Is Associated With Excess Superoxide Formation.” American Journal of Physiology. Renal Physiology 289, no. 2: F420–F430. 15814529 10.1152/ajprenal.00415.2004

[jnc70529-bib-0115] Ryle, C. , C. K. Leow , and M. Donaghy . 1997. “Nonenzymatic Glycation of Peripheral and Central Nervous System Proteins in Experimental Diabetes Mellitus.” Muscle & Nerve 20, no. 5: 577–584. 9140364 10.1002/(sici)1097-4598(199705)20:5<577::aid-mus6>3.0.co;2-6

[jnc70529-bib-0116] Santiago, J. V. , P. H. Snksen , A. J. Boulton , et al. 1993. “Withdrawal of the Aldose Reductase Inhibitor Tolrestat in Patients With Diabetic Neuropathy: Effect on Nerve Function. The Tolrestat Study Group.” Journal of Diabetes and Its Complications 7, no. 3: 170–178. 8343611 10.1016/1056-8727(93)90042-w

[jnc70529-bib-0117] Saraei, P. , I. Asadi , M. A. Kakar , and N. Moradi‐Kor . 2019. “The Beneficial Effects of Metformin on Cancer Prevention and Therapy: A Comprehensive Review of Recent Advances.” Cancer Management and Research 11: 3295–3313. 31114366 10.2147/CMAR.S200059 PMC6497052

[jnc70529-bib-0118] Sasaki, Y. 2019. “Metabolic Aspects of Neuronal Degeneration: From a NAD(+) Point of View.” Neuroscience Research 139: 9–20. 30006197 10.1016/j.neures.2018.07.001 PMC6674977

[jnc70529-bib-0119] Sato‐Yamada, Y. , A. Strickland , Y. Sasaki , J. Bloom , A. DiAntonio , and J. Milbrandt . 2022. “A SARM1‐Mitochondrial Feedback Loop Drives Neuropathogenesis in a Charcot‐Marie‐Tooth Disease Type 2A Rat Model.” Journal of Clinical Investigation 132, no. 23: e161566. 36287202 10.1172/JCI161566 PMC9711878

[jnc70529-bib-0120] Schalkwijk, C. G. , and C. D. A. Stehouwer . 2020. “Methylglyoxal, a Highly Reactive Dicarbonyl Compound, in Diabetes, Its Vascular Complications, and Other Age‐Related Diseases.” Physiological Reviews 100, no. 1: 407–461. 31539311 10.1152/physrev.00001.2019

[jnc70529-bib-0121] Schmeichel, A. M. , J. D. Schmelzer , and P. A. Low . 2003. “Oxidative Injury and Apoptosis of Dorsal Root Ganglion Neurons in Chronic Experimental Diabetic Neuropathy.” Diabetes 52, no. 1: 165–171. 12502508 10.2337/diabetes.52.1.165

[jnc70529-bib-0122] Sekhar, R. V. , S. V. McKay , S. G. Patel , et al. 2011. “Glutathione Synthesis Is Diminished in Patients With Uncontrolled Diabetes and Restored by Dietary Supplementation With Cysteine and Glycine.” Diabetes Care 34, no. 1: 162–167. 20929994 10.2337/dc10-1006 PMC3005481

[jnc70529-bib-0123] Semba, R. D. , E. J. Nicklett , and L. Ferrucci . 2010. “Does Accumulation of Advanced Glycation End Products Contribute to the Aging Phenotype?” Journals of Gerontology. Series A, Biological Sciences and Medical Sciences 65, no. 9: 963–975. 20478906 10.1093/gerona/glq074 PMC2920582

[jnc70529-bib-0124] Seo, K. , S. H. Ki , and S. M. Shin . 2014. “Methylglyoxal Induces Mitochondrial Dysfunction and Cell Death in Liver.” Toxicology Research 30, no. 3: 193–198. 10.5487/TR.2014.30.3.193 PMC4206746 25343013

[jnc70529-bib-0125] Seryogina, E. S. , A. V. Kamynina , D. O. Koroev , O. M. Volpina , A. Y. Vinokurov , and A. Y. Abramov . 2024. “RAGE Induces Physiological Activation of NADPH Oxidase in Neurons and Astrocytes and Neuroprotection.” FEBS Journal 291, no. 9: 1944–1957. 38335056 10.1111/febs.17086

[jnc70529-bib-0126] Shibanuma, M. , A. Inoue , K. Ushida , et al. 2011. “Importance of Mitochondrial Dysfunction in Oxidative Stress Response: A Comparative Study of Gene Expression Profiles.” Free Radical Research 45, no. 6: 672–680. 21391894 10.3109/10715762.2011.564169

[jnc70529-bib-0127] Silhavy, J. , H. Malinska , M. Huttl , et al. 2020. “Downregulation of the Glo1 Gene Is Associated With Reduced Adiposity and Ectopic Fat Accumulation in Spontaneously Hypertensive Rats.” Antioxidants 9, no. 12: 1179. 33255888 10.3390/antiox9121179 PMC7759780

[jnc70529-bib-0128] Sima, A. A. , D. A. Greene , M. B. Brown , et al. 1993. “Effect of Hyperglycemia and the Aldose Reductase Inhibitor Tolrestat on Sural Nerve Biochemistry and Morphometry in Advanced Diabetic Peripheral Polyneuropathy. The Tolrestat Study Group.” Journal of Diabetes and Its Complications 7, no. 3: 157–169. 8343610 10.1016/1056-8727(93)90041-v

[jnc70529-bib-0129] Simpson, D. M. , J. Robinson‐Papp , J. Van , et al. 2017. “Capsaicin 8% Patch in Painful Diabetic Peripheral Neuropathy: A Randomized, Double‐Blind, Placebo‐Controlled Study.” Journal of Pain 18, no. 1: 42–53. 27746370 10.1016/j.jpain.2016.09.008

[jnc70529-bib-0130] Slouma, M. , S. Ben Dhia , E. Cheour , and I. Gharsallah . 2024. “Acroparesthesias: An Overview.” Current Rheumatology Reviews 20, no. 2: 115–126. 37921132 10.2174/0115733971254976230927113202

[jnc70529-bib-0131] Soulis, T. , M. E. Cooper , D. Vranes , R. Bucala , and G. Jerums . 1996. “Effects of Aminoguanidine in Preventing Experimental Diabetic Nephropathy Are Related to the Duration of Treatment.” Kidney International 50, no. 2: 627–634. 8840295 10.1038/ki.1996.358

[jnc70529-bib-0132] Staehelin Jensen, T. 2023. “The Pathogenesis of Painful Diabetic Neuropathy and Clinical Presentation.” Diabetes Research and Clinical Practice 206, no. Suppl 1: 110753. 38245319 10.1016/j.diabres.2023.110753

[jnc70529-bib-0133] Stinson, S. E. , A. A. Shadrin , Z. Rahman , et al. 2025. “Distinct Metabolic Signatures of Alzheimer's and Parkinson's Disease Revealed Through Genetic Overlap With Metabolic Markers.” medRxiv 127: 2025.07.31.25332114. 10.1016/j.ebiom.2026.106254 PMC13091360 41966729

[jnc70529-bib-0134] Stratmann, B. , B. Goldstein , P. J. Thornalley , N. Rabbani , and D. Tschoepe . 2017. “Intracellular Accumulation of Methylglyoxal by Glyoxalase 1 Knock Down Alters Collagen Homoeostasis in L6 Myoblasts.” International Journal of Molecular Sciences 18, no. 3: 480. 28241483 10.3390/ijms18030480 PMC5372496

[jnc70529-bib-0135] Strom, A. , K. Strassburger , M. Schmuck , et al. 2021. “Interaction Between Magnesium and Methylglyoxal in Diabetic Polyneuropathy and Neuronal Models.” Molecular Metabolism 43: 101114. 33166742 10.1016/j.molmet.2020.101114 PMC7704399

[jnc70529-bib-0136] Suantawee, T. , T. Thilavech , H. Cheng , and S. Adisakwattana . 2020. “Cyanidin Attenuates Methylglyoxal‐Induced Oxidative Stress and Apoptosis in INS‐1 Pancreatic Beta‐Cells by Increasing Glyoxalase‐1 Activity.” Nutrients 12, no. 5: 1319. 32384625 10.3390/nu12051319 PMC7284759

[jnc70529-bib-0137] Tan, D. , Y. Wang , C. Y. Lo , S. Sang , and C. T. Ho . 2008. “Methylglyoxal: Its Presence in Beverages and Potential Scavengers.” Annals of the New York Academy of Sciences 1126: 72–75. 18448797 10.1196/annals.1433.027

[jnc70529-bib-0138] Tesfaye, S. , G. Sloan , J. Petrie , et al. 2022. “Comparison of Amitriptyline Supplemented With Pregabalin, Pregabalin Supplemented With Amitriptyline, and Duloxetine Supplemented With Pregabalin for the Treatment of Diabetic Peripheral Neuropathic Pain (OPTION‐DM): A Multicentre, Double‐Blind, Randomised Crossover Trial.” Lancet 400, no. 10353: 680–690. 36007534 10.1016/S0140-6736(22)01472-6 PMC9418415

[jnc70529-bib-0139] Thornalley, P. J. 2002. “Glycation in Diabetic Neuropathy: Characteristics, Consequences, Causes, and Therapeutic Options.” International Review of Neurobiology 50: 37–57. 12198817 10.1016/s0074-7742(02)50072-6

[jnc70529-bib-0140] Thornalley, P. J. 2003. “Use of Aminoguanidine (Pimagedine) to Prevent the Formation of Advanced Glycation Endproducts.” Archives of Biochemistry and Biophysics 419, no. 1: 31–40. 14568006 10.1016/j.abb.2003.08.013

[jnc70529-bib-0141] Thornalley, P. J. 2008. “Protein and Nucleotide Damage by Glyoxal and Methylglyoxal in Physiological Systems–Role in Ageing and Disease.” Drug Metabolism and Drug Interactions 23, no. 1–2: 125–150. 18533367 10.1515/dmdi.2008.23.1-2.125 PMC2649415

[jnc70529-bib-0142] Tobon‐Velasco, J. C. , E. Cuevas , and M. A. Torres‐Ramos . 2014. “Receptor for AGEs (RAGE) as Mediator of NF‐kB Pathway Activation in Neuroinflammation and Oxidative Stress.” CNS & Neurological Disorders Drug Targets 13, no. 9: 1615–1626. 25106630 10.2174/1871527313666140806144831

[jnc70529-bib-0143] Tolrestat . 2005. “DB02383.” https://go.drugbank.com/drugs/DB02383.

[jnc70529-bib-0144] Ueno, T. , M. Yamanaka , W. Taniguchi , et al. 2024. “Methylglyoxal Activates Transient Receptor Potential A1/V1 via Reactive Oxygen Species in the Spinal Dorsal Horn.” Molecular Pain 20: 17448069241233744. 38323375 10.1177/17448069241233744 PMC10868495

[jnc70529-bib-0145] Uribarri, J. , S. Woodruff , S. Goodman , et al. 2010. “Advanced Glycation End Products in Foods and a Practical Guide to Their Reduction in the Diet.” Journal of the American Dietetic Association 110, no. 6: 911‐16. 20497781 10.1016/j.jada.2010.03.018 PMC3704564

[jnc70529-bib-0146] Vafaeinasab, M. , F. Mirzaei Malekabad , A. Khatibi , A. Ghadiri‐Anari , M. Zare Bidaki , and R. Azizi . 2025. “The Comparison of the Effect of Adding Empagliflozin to the Medication Regimen on Peripheral Neuropathy in Patients With Type II Diabetes: A Double Blind Randomised Clinical Trial.” Endocrinology, Diabetes & Metabolism 8, no. 6: e70128. 10.1002/edm2.70128 PMC12634468 41267217

[jnc70529-bib-0147] Vicente Miranda, H. , M. A. Gomes , J. Branco‐Santos , et al. 2016. “Glycation Potentiates Neurodegeneration in Models of Huntington's Disease.” Scientific Reports 6: 36798. 27857176 10.1038/srep36798 PMC5114697

[jnc70529-bib-0148] Vlassara, H. , and J. Uribarri . 2014. “Advanced Glycation End Products (AGE) and Diabetes: Cause, Effect, or Both?” Current Diabetes Reports 14, no. 1: 453. 24292971 10.1007/s11892-013-0453-1 PMC3903318

[jnc70529-bib-0149] Vulesevic, B. , B. McNeill , F. Giacco , et al. 2016. “Methylglyoxal‐Induced Endothelial Cell Loss and Inflammation Contribute to the Development of Diabetic Cardiomyopathy.” Diabetes 65, no. 6: 1699–1713. 26956489 10.2337/db15-0568 PMC4878427

[jnc70529-bib-0150] Vulesevic, B. , R. W. Milne , and E. J. Suuronen . 2014. “Reducing Methylglyoxal as a Therapeutic Target for Diabetic Heart Disease.” Biochemical Society Transactions 42, no. 2: 523–527. 24646272 10.1042/BST20130254

[jnc70529-bib-0151] Wahren, J. , H. Foyt , M. Daniels , and J. C. Arezzo . 2016. “Long‐Acting C‐Peptide and Neuropathy in Type 1 Diabetes: A 12‐Month Clinical Trial.” Diabetes Care 39, no. 4: 596–602. 26884473 10.2337/dc15-2068

[jnc70529-bib-0152] Wang, G. , Y. Wang , Q. Yang , et al. 2022. “Metformin Prevents Methylglyoxal‐Induced Apoptosis by Suppressing Oxidative Stress In Vitro and In Vivo.” Cell Death & Disease 13, no. 1: 29. 35013107 10.1038/s41419-021-04478-x PMC8748764

[jnc70529-bib-0153] Wang, H. , S. Boeren , W. Bakker , I. Rietjens , E. Saccenti , and L. Zheng . 2024. “An Integrated Proteomics and Metabolomics Analysis of Methylglyoxal‐Induced Neurotoxicity in a Human Neuroblastoma Cell Line.” NPJ Science of Food 8, no. 1: 84. 39448607 10.1038/s41538-024-00328-0 PMC11502746

[jnc70529-bib-0154] Wang, Y. , J. Chen , Y. Zheng , et al. 2024. “Glucose Metabolite Methylglyoxal Induces Vascular Endothelial Cell Pyroptosis via NLRP3 Inflammasome Activation and Oxidative Stress In Vitro and In Vivo.” Cellular and Molecular Life Sciences 81, no. 1: 401. 39269632 10.1007/s00018-024-05432-8 PMC11399538

[jnc70529-bib-0155] Wang, Z. , S. Liu , M. Zhang , and M. Liu . 2025. “Dual Roles of Methylglyoxal in Cancer.” Frontiers in Oncology 15: 1557162. 40352588 10.3389/fonc.2025.1557162 PMC12061732

[jnc70529-bib-0156] Wautier, M. P. , O. Chappey , S. Corda , D. M. Stern , A. M. Schmidt , and J. L. Wautier . 2001. “Activation of NADPH Oxidase by AGE Links Oxidant Stress to Altered Gene Expression via RAGE.” American Journal of Physiology. Endocrinology and Metabolism 280, no. 5: E685–E694. 11287350 10.1152/ajpendo.2001.280.5.E685

[jnc70529-bib-0157] Willemen, H. , P. S. Santos Ribeiro , M. Broeks , et al. 2023. “Inflammation‐Induced Mitochondrial and Metabolic Disturbances in Sensory Neurons Control the Switch From Acute to Chronic Pain.” Cell Reports Medicine 4, no. 11: 101265. 37944527 10.1016/j.xcrm.2023.101265 PMC10694662

[jnc70529-bib-0158] Xue, M. , N. Rabbani , and P. J. Thornalley . 2025. “Glyoxalase 1 Inducer, Trans‐Resveratrol and Hesperetin‐Dietary Supplement With Multi‐Modal Health Benefits.” Antioxidants (Basel) 14, no. 8: 956. 40867852 10.3390/antiox14080956 PMC12382748

[jnc70529-bib-0159] Yamagishi, S. , T. Matsui , and K. Fukami . 2015. “Role of Receptor for Advanced Glycation End Products (RAGE) and Its Ligands in Cancer Risk.” Rejuvenation Research 18, no. 1: 48–56. 25472493 10.1089/rej.2014.1625

[jnc70529-bib-0160] Yang, Z. , W. Zhang , H. Lu , and S. Cai . 2022. “Methylglyoxal in the Brain: From Glycolytic Metabolite to Signalling Molecule.” Molecules 27, no. 22: 7905. 36432007 10.3390/molecules27227905 PMC9696358

[jnc70529-bib-0161] Yoo, H. J. , C. O. Hong , S. K. Ha , and K. W. Lee . 2020. “Chebulic Acid Prevents Methylglyoxal‐Induced Mitochondrial Dysfunction in INS‐1 Pancreatic Beta‐Cells.” Antioxidants 9, no. 9: 771. 32825285 10.3390/antiox9090771 PMC7554990

[jnc70529-bib-0162] Yousuf, M. S. , M. Mancilla Moreno , B. J. Woodall , et al. 2025. “Diroximel Fumarate Acts Through Nrf2 to Attenuate Methylglyoxal‐Induced Nociception in Mice and Decrease ISR Activation in DRG Neurons.” Diabetes 74, no. 5: 827–837. 39976640 10.2337/db23-1025 PMC12015141

[jnc70529-bib-0163] Yumnam, S. , L. Subedi , and S. Y. Kim . 2020. “Glyoxalase System in the Progression of Skin Aging and Skin Malignancies.” International Journal of Molecular Sciences 22, no. 1: 310. 33396745 10.3390/ijms22010310 PMC7794849

[jnc70529-bib-0164] Zgutka, K. , M. Tkacz , P. Tomasiak , and M. Tarnowski . 2023. “A Role for Advanced Glycation End Products in Molecular Ageing.” International Journal of Molecular Sciences 24, no. 12: 9881. 37373042 10.3390/ijms24129881 PMC10298716

[jnc70529-bib-0165] Zhou, H. , L. Wang , Z. Lin , et al. 2023. “Methylglyoxal From Gut Microbes Boosts Radiosensitivity and Radioimmunotherapy in Rectal Cancer by Triggering Endoplasmic Reticulum Stress and cGAS‐STING Activation.” Journal for Immunotherapy of Cancer 11, no. 11: e007840. 38035726 10.1136/jitc-2023-007840 PMC10689421

[jnc70529-bib-0166] Ziegler, D. , P. A. Low , W. J. Litchy , et al. 2011. “Efficacy and Safety of Antioxidant Treatment With Alpha‐Lipoic Acid Over 4 Years in Diabetic Polyneuropathy: The NATHAN 1 Trial.” Diabetes Care 34, no. 9: 2054–2060. 21775755 10.2337/dc11-0503 PMC3161301

